# Redefining Obstructive Sleep Apnea: Treatment in the Modern Era

**DOI:** 10.3390/pathophysiology33010020

**Published:** 2026-03-02

**Authors:** Jose Redondo, Kori B. Ascher, Alexandre R. Abreu

**Affiliations:** 1 Internal Medicine, Jackson Memorial Hospital and University of Miami, 1611 NW 12th Ave, Miami, FL 33136, USA; jxr4676@miami.edu; 2 Miller School of Medicine, 1600 NW 10th Ave, Miami, FL 33136, USA; 3 Division of Pulmonary, Critical Care, and Sleep Medicine, University of Miami, 1611 NW 12th Ave, Miami, FL 33136, USA

**Keywords:** obstructive sleep apnea, CPAP adherence, mandibular advancement devices, hypoglossal nerve stimulation, maxillomandibular advancement, positional therapy, neuromuscular electrical stimulation, carbonic anhydrase inhibitors, tirzepatide and weight loss

## Abstract

Background: Obstructive sleep apnea (OSA) is a highly prevalent and heterogeneous disorder associated with substantial cardiometabolic morbidity. Although continuous positive airway pressure (CPAP) remains first-line therapy, long-term effectiveness is frequently limited by suboptimal adherence. Advances in airway devices, surgical techniques, neuromodulation, and pharmacologic therapies have expanded the therapeutic landscape and created opportunities for individualized, mechanism-based treatment. Methods: We conducted a selective, narrative review with structured quantitative synthesis of randomized controlled trials, comparative cohorts, long-term follow-up studies, registries, and mechanistic investigations addressing OSA therapies beyond CPAP. Evidence spanning oral appliances, upper-airway and skeletal surgery, hypoglossal nerve stimulation, neuromuscular electrical stimulation, positional therapy, and pharmacologic interventions targeting metabolic and non-anatomical endotypes was integrated. Outcomes of interest included apnea–hypopnea index (AHI), oxygenation, blood pressure, patient-reported symptoms, durability, safety, and real-world adherence. Results: Mandibular advancement devices (MADs) consistently reduced AHI relative to placebo and produced symptom relief comparable to CPAP in mild-to-moderate OSA, largely due to superior adherence. Palatal surgery yielded meaningful short-term improvement in selected patients but demonstrated limited long-term durability. In contrast, maxillomandibular advancement (MMA) achieved the largest and most durable reductions in OSA severity, with efficacy comparable to CPAP and superior to other surgical modalities in appropriate skeletal phenotypes. Hypoglossal nerve stimulation (HNS) produced substantial, durable improvements in AHI and symptoms with high adherence, supported by randomized trials, long-term follow-up, and real-world registry data; newer bilateral and proximal stimulation systems may further broaden candidacy. Neuromuscular electrical stimulation and positional therapy provided modest, phenotype-dependent benefits, primarily as adjunctive or early-stage interventions. A major advance is the emergence of metabolic and endotype-targeted pharmacotherapy: longitudinal data demonstrate a dose-dependent relationship between weight change and OSA progression or regression, while randomized trials show that GLP-1-based therapies—particularly dual GLP-1/GIP agonism with tirzepatide—produce large, clinically meaningful reductions in AHI and cardiometabolic risk in obesity-associated OSA. Additional pharmacologic strategies targeting ventilatory loop gain and arousal threshold further support an endotype-driven treatment paradigm. Conclusions: Contemporary OSA management is shifting from a CPAP-centric model toward a precision-guided, multimodal framework that aligns therapy with dominant anatomic and physiological contributors to airway collapse. Integrating metabolic, neuromodulatory, and structural interventions—often in combination—offers the potential for durable disease control and improved patient-centered outcomes. Future priorities include head-to-head and combination trials, long-term cardiovascular outcomes, cost-effectiveness analyses, and pragmatic tools to operationalize personalized OSA therapy in routine clinical practice.

## 1. Introduction

Obstructive sleep apnea (OSA) is a prevalent chronic sleep-related breathing disorder, affecting approximately 13% of men and 6% of women aged 30–70 in the U.S. [1]. It is characterized by recurrent episodes of upper airway obstruction during sleep, resulting in apneas or hypopneas that lead to intermittent hypoxemia, sleep fragmentation, and recurrent arousals. OSA has been associated with excessive daytime sleepiness, cognitive impairment, mood disturbance, and reduced quality of life, as well as an increased risk of hypertension, cardiovascular events, type 2 diabetes, and all-cause mortality [2,3,4]. Given its chronic nature and potential serious health consequences, effective and sustainable long-term management therapies are essential.

Continuous positive airway pressure (CPAP) remains the first-line therapy for OSA. When used consistently, CPAP acts by splinting the upper airway and preventing its collapse, thereby improving daytime sleepiness, neurocognitive performance, and blood pressure regulation [5,6]. In patients with severe OSA, effective CPAP treatment has been shown to reduce the risk of cardiovascular events; however, its real-world effectiveness is often limited by suboptimal adherence to therapy. Studies indicate that approximately 25–50% of patients discontinue CPAP therapy within the first year, and even among those who continue, the average nightly use frequently falls below the recommended duration of 4 h per night [7,8]. Overall, between 46% and 83% of OSA patients are considered non-adherent to CPAP therapy [9]. In the SAVE trial, the mean CPAP use among participants with cardiovascular disease was approximately three h per night [6]. Despite these adherence challenges, when CPAP is utilized diligently, it effectively normalizes the AHI, alleviates symptoms, improves quality of life, and may reduce cardiovascular risk in patients with severe OSA [4,6].

This persistent challenge with adherence leaves a substantial proportion of patients either untreated or inadequately managed. Given that OSA is a chronic condition necessitating lifelong therapy, many individuals ultimately fail or discontinue CPAP therapy, prompting the need for alternative treatment options. Over the past decade, significant advancements have emerged across multiple domains, including oral appliance therapy, innovative surgical and neurostimulation approaches, daytime training interventions, positional therapy, and novel pharmacologic strategies targeting OSA pathophysiology (Table 1).

This review provides a comprehensive and up-to-date analysis of recent progress in OSA management. We first address the current status and limitations of positive airway pressure (PAP) therapy, followed by an appraisal of evidence supporting mandibular advancement devices (MADs), surgical interventions, neuromuscular electrical stimulation therapies, and novel modalities such as tongue-base cryotherapy and positional devices. Additionally, we evaluate emerging pharmacotherapies ranging from sedative agents designed to raise the arousal threshold to carbonic anhydrase inhibitors aimed at lowering ventilatory loop gain, as well as metabolic treatments addressing obesity. Throughout this review, we compare efficacy data from pivotal randomized controlled trials, examine adherence and tolerability considerations, and identify persisting gaps in evidence. Collectively, these developments reflect a broader shift in OSA management from a historically CPAP-centric model toward a more personalized, multimodal framework aligned with underlying pathophysiology. Contemporary clinical practice guidelines and systematic reviews increasingly recognize oral appliances, hypoglossal nerve stimulation, surgical interventions, positional therapy, and pharmacologic strategies as complementary components of OSA management in appropriately selected patients, rather than simply alternatives reserved for CPAP failure [10,11,12]. Moreover, advances in endotype characterization, including the roles of upper-airway anatomy, ventilatory loop gain, and arousal threshold, have provided a mechanistic basis for phenotype-guided therapy selection [13,14]. Together, these developments support a precision-based approach in which treatment is matched to dominant pathophysiologic contributors rather than relying exclusively on positive airway pressure therapy. Future research directions and clinical recommendations are discussed to enhance long-term treatment adherence and optimize patient outcomes.

**Table 1:** Comparative overview of major therapeutic modalities for adult obstructive sleep apnea. Therapies are organized by primary mechanism of action, typical adult candidate profile, adherence considerations, and key limitations. The table provides a structured comparative framework reflecting mechanism-based and phenotype-guided treatment selection rather than a hierarchical ranking of efficacy [11,12,15,16,17,18,19,20,21,22,23].

Therapy	Primary Mechanism of Action	Typical Candidate Profile	Adherence Considerations	Key Limitations
Continuous Positive Airway Pressure (CPAP)	Pneumatic splinting of the upper airway via continuous positive intraluminal pressure, preventing dynamic collapse throughout the respiratory cycle.	Adults with moderate–to–severe OSA across phenotypes; first-line therapy in most guidelines.	Requires consistent nightly use; adherence limited by mask discomfort, pressure intolerance, and nasal symptoms.	High physiologic efficacy but real-world effectiveness limited by adherence.
Mandibular Advancement Device (MAD)	Anterior mandibular advancement increases retropalatal and retrolingual airway caliber, reducing pharyngeal collapsibility.	Mild–to–moderate OSA; CPAP-intolerant patients; favorable craniofacial anatomy (e.g., retrognathia, positional OSA).	Generally well tolerated; requires titration and dental follow-up.	Reduced efficacy in severe OSA or obesity; residual AHI common in advanced disease.
Tonsillectomy (Adult)	Removal of hypertrophic palatine tonsils reduces lateral pharyngeal wall obstruction and enlarges the retropalatal airway.	Adults with marked tonsillar hypertrophy and favorable airway anatomy; CPAP-intolerant selected cases.	Short-term postoperative pain and dysphagia.	Highly anatomy-dependent; limited benefit without significant tonsillar enlargement.
Palatal Surgery (e.g., UPPP, ESP)	Resection and/or repositioning of palatal tissues reduces retropalatal obstruction and lateral wall collapse.	CPAP-intolerant adults with palatal-dominant obstruction.	Postoperative recovery and pain control required.	Variable efficacy; limited long-term durability; irreversible anatomical changes.
Maxillomandibular Advancement (MMA)	Skeletal advancement enlarges retropalatal and retrolingual airway and increases longitudinal airway tension.	Severe OSA with craniofacial restriction; CPAP intolerance; multilevel anatomic obstruction.	Major surgery with recovery and orthodontic coordination.	High upfront invasiveness; limited availability; surgical risk.
Hypoglossal Nerve Stimulation (HNS)	Inspiratory-synchronized stimulation of the hypoglossal nerve advances and stabilizes the tongue.	Moderate–to–severe OSA; CPAP-intolerant; non-obese; no concentric palatal collapse.	High adherence after implantation; requires device activation and follow-up.	High upfront cost; strict eligibility criteria; limited > 5-year data.
Neuromuscular Electrical Stimulation (NMES)	Noninvasive electrical activation of tongue muscles increases upper airway tone.	Mild OSA or residual symptoms; adjunctive therapy.	Daily use required; adherence variable.	Modest efficacy; limited long-term data.
Positional Therapy	Reduces supine sleep to minimize position-dependent airway collapse.	Positional OSA; lower BMI; mild disease.	Requires nightly device use or behavioral reinforcement.	Limited benefit in non-positional or severe OSA.
Nasal Expiratory Positive Airway Pressure (EPAP)	Expiratory resistance generates positive end-expiratory pressure, stabilizing the upper airway.	Mild–moderate OSA; travel/backup therapy; adequate nasal patency required.	Nightly use of single-use valves.	Reduced efficacy in severe OSA; nasal obstruction limits tolerance.
Pharmacologic Therapy (Endotype-Targeted)	Modulates metabolic load (GLP-1/GIP), ventilatory loop gain (CAIs), or arousal threshold (eszopiclone).	Obesity-predominant OSA or physiologic endotypes (high loop gain, low arousal threshold).	Continuous therapy required; monitoring for adverse effects.	Incomplete normalization as monotherapy; long-term safety data limited.

## 2. Methods

### 2.1. Review Design

This article was conducted as a selective, narrative review with structured quantitative synthesis focused on contemporary therapies for obstructive sleep apnea (OSA). Given the diversity of interventions reviewed—including positive airway pressure, oral appliance therapy, surgical procedures, neuromodulation, pharmacotherapy, and adjunctive noninvasive therapies—as well as substantial heterogeneity in study design, outcome definitions, and follow-up duration, a formal systematic review or meta-analysis was not performed. Instead, a transparent, mechanism-driven approach was used to integrate clinically and pathophysiologically relevant evidence.

### 2.2. Literature Search

A literature search was performed using PubMed/MEDLINE, EMBASE, and Google Scholar to identify relevant studies published between January 2000 and March 2025. Searches were limited to English-language, peer-reviewed articles involving adult populations.

Search terms were applied alone and in combination and included:

“obstructive sleep apnea,” “continuous positive airway pressure,” “mandibular advancement device,” “oral appliance therapy,” “uvulopalatopharyngoplasty,” “upper airway surgery,” “maxillomandibular advancement,” “hypoglossal nerve stimulation,” “upper airway stimulation,” “neuromuscular electrical stimulation,” “positional therapy,” “loop gain,” and “arousal threshold.”

In addition, the reference lists of key randomized trials, long-term follow-up studies, large registries, and recent systematic reviews were manually reviewed to identify additional relevant publications.

### 2.3. Study Selection

Titles and abstracts were reviewed independently by two authors to identify studies relevant to treatment efficacy, durability, adherence, safety, or patient-selection considerations in OSA. Full-text review was performed for articles meeting the inclusion criteria. Disagreements regarding study inclusion were resolved through discussion and consensus.

### 2.4. Eligibility Criteria

Studies were considered eligible if they met one or more of the following criteria: Randomized or controlled clinical trials evaluating OSA therapies against sham, placebo, medical management, or active comparators. Prospective or retrospective cohort studies reporting objective sleep outcomes such as the apnea–hypopnea index (AHI) or oxygen desaturation index (ODI). Long-term follow-up studies or clinical registries assessing durability, adherence, or real-world effectiveness. Systematic reviews or meta-analyses providing contextual evidence regarding efficacy or safety.

Only studies involving adult populations (≥18 years) were included in this review. Studies focused exclusively on pediatric obstructive sleep apnea were excluded due to distinct pathophysiology and treatment algorithms. Case reports or small uncontrolled series without objective sleep metrics, or those that were not peer-reviewed, were also excluded.

### 2.5. Data Extraction

From each included study, the following information was extracted when available: study design and population characteristics, sample size and comparator, baseline and follow-up AHI and/or ODI, patient-reported outcomes (including the Epworth Sleepiness Scale and Functional Outcomes of Sleep Questionnaire), responder definitions and response rates, adherence metrics, duration of follow-up, and adverse events.

Data extraction emphasized clinically interpretable outcomes relevant to treatment selection and long-term disease control.

### 2.6. Quantitative Synthesis

Quantitative results were summarized using effect measures appropriate to the study design, without statistical pooling across heterogeneous interventions.

Within-group changes (e.g., absolute or relative reductions in AHI or ODI, responder proportions) were reported for single-arm studies, cohorts, and registries. Between-group comparative measures (including risk ratios, odds ratios, absolute risk differences, and number needed to treat) were calculated only for randomized studies with concurrent control groups. Standardized mean differences were reported when provided by the original studies, but were not recalculated.

Formal meta-analysis was not undertaken due to heterogeneity in therapeutic modalities, outcome definitions, and follow-up intervals.

### 2.7. Assessment of Heterogeneity and Bias

Clinical and methodological heterogeneity was addressed through stratification by therapeutic modality and by distinguishing randomized comparative evidence from observational and long-term durability data. Potential sources of bias, including selection bias, attrition, and confounding in nonrandomized studies, are acknowledged where relevant. Systematic reviews and meta-analyses were used to contextualize findings, but were not pooled with primary studies to avoid duplication.

### 2.8. Conceptual Framework

Findings were interpreted using a pathophysiology-based, precision-medicine framework, linking treatment mechanisms to dominant contributors to airway obstruction, including anatomic collapse, neuromuscular responsiveness, ventilatory control instability, arousal threshold, and obesity-related mechanical load. This framework informed cross-modality comparisons and the proposed treatment-selection algorithm.

## 3. Mandibular Advancement Devices (MADs)

**Figure 1:**
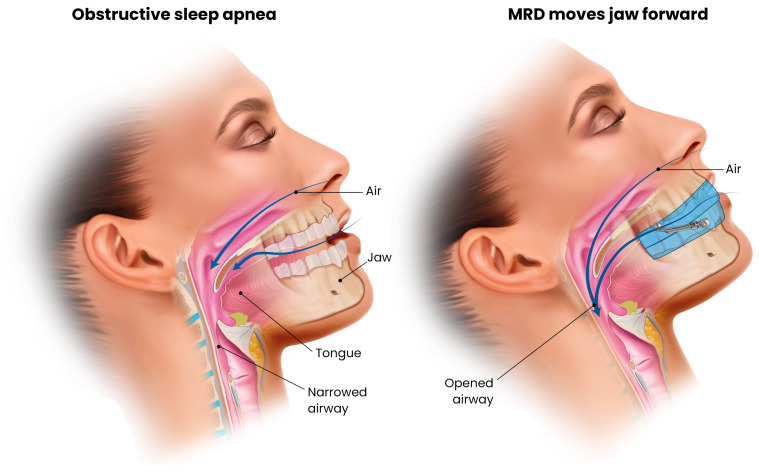
Effect of mandibular advancement device on upper airway anatomy. Sagittal schematic illustrating upper airway narrowing during obstructive sleep apnea (**left**) and airway enlargement with mandibular advancement device therapy (**right**). Forward advancement of the mandible and tongue increases retropalatal and retrolingual airway caliber, reducing upper airway collapsibility and improving airflow during sleep.

Mandibular advancement devices are custom-fitted oral appliances designed to advance the mandible during sleep, thereby enlarging and stabilizing the upper airway. By anteriorly displacing the retroglossal and retropalatal airway caliber and subsequently reducing the propensity of upper airway collapse (Figure 1). MADs are typically indicated for patients with mild to moderate OSA or for those who are unable to tolerate CPAP therapy. Over the past two decades, they have become an established second-line treatment supported by substantial clinical and physiological evidence (Table 2).

During sleep, MADs advance the mandible to approximately 50–75% of the individual’s maximal protrusion, and some designs include components that simultaneously advance the tongue [24]. This anterior traction of the tongue and hyoid musculature enlarges the pharyngeal airway [24]. By repositioning the jaw forward, MADs also tense the soft tissues attached to the mandible, including the tongue, palatal muscles, and lateral pharyngeal walls [25]. This results in an enlarged retroglossal airway space, with reduced collapsibility during sleep. Imaging and endoscopic studies demonstrate increased velopharyngeal cross-sectional area and reduced pharyngeal compliance with a MAD in place [26].

In contrast to CPAP, which pneumatically splints the airway open, MADs act by mechanically and anatomically modifying upper airway geometry during sleep. Therapeutic response varies depending on craniofacial structure and site of obstruction. MADs are generally most effective in patients with positional OSA or retrognathia with tongue base collapse [27,28,29]. Contemporary devices are titratable, allowing gradual mandibular advancement to optimize efficacy while minimizing jaw discomfort. Additionally, many patients and bed partners report improved comfort and satisfaction with MADs compared with CPAP, owing to the absence of noise, tubing, and facial masks, as well as greater portability for travel [30].

Across the literature, clinical trials have consistently demonstrated that MAD therapy significantly reduces the severity of OSA compared with placebo or non-advancing devices, and, in many cases, approaches the effectiveness of CPAP for mild to moderate disease [31,32]. While CPAP remains superior in its ability to reduce the AHI across all severities, MAD therapy frequently produces clinically meaningful improvements and achieves comparable symptom relief [33,34,35,36].

Across controlled trials comparing MAD with CPAP, CPAP consistently achieved greater improvement in objective respiratory indices, with MAD associated with higher residual AHI or ODI (MAD − CPAP differences ranging approximately +6 to +9 events/h for AHI and −11.4 events/h for ODI when reported as CPAP superiority). In contrast, subjective sleepiness outcomes were broadly similar between therapies, with small and inconsistent differences in ESS.

**Table 2:** Mandibular advancement device (MAD) therapy: quantitative comparison versus CPAP and placebo controls across multiple trials [33,34,35,36,37,38]. Outcomes include objective indices of OSA severity (AHI, ODI) and subjective daytime sleepiness (Epworth Sleepiness Scale). Mean differences are presented with standardized directionality (MAD − comparator) to allow rapid comparison across therapies and study designs. Where crossover trials did not report paired treatment effect variance (or the paired confidence interval), estimates are presented without 95% confidence intervals to avoid assumptions about within-subject correlation. For AHI/ODI, negative values indicate improvement with MAD; positive values favor the comparator. For ESS, negative values indicate less sleepiness with MAD.

Study	Design	N	Comparator	Outcome	Time Point	Effect Estimate (MAD − Comparator)	Interpretation
Barnes, M. et al. [34]	Randomized three-way crossover trial	99	CPAP	AHI (events/h)	3 months	+9.2 (CI not reported †)	CPAP superior (lower AHI)
Barnes, M. et al. [34]	Randomized three-way crossover trial	99	CPAP	ESS (points)	3 months	0.0 (CI not reported †)	No difference
Phillips, C. L. et al. [36]	Randomized crossover RCT	126	CPAP	AHI (events/h)	1 month	+6.6 (CI not reported †)	CPAP superior
Phillips, C. L. et al. [36]	Randomized crossover RCT	126	CPAP	ESS (points)	1 month	−0.31 (95% CI −0.90 to +0.20) *	No difference
Hamoda, M. M. et al. [37]	Double-randomised, three-phase trial	81	CPAP	ODI (events/h)	6 months	−11.4 (95% CI −14.9 to −7.9) **	CPAP superior (greater ODI reduction)
Ferguson, K. A. et al. [38]	Randomized crossover RCT	27	CPAP	AHI (events/h)	4 months	+6.1 (CI not reported †)	CPAP superior
Barnes, M. et al. [34]	Randomized three-way crossover RCT	99	Placebo	AHI (events/h)	3 months	−6.3 (CI not reported ‡)	MAD superior vs. sham
Barnes, M. et al. [34]	Randomized three-way crossover RCT	99	Placebo	ESS (points)	3 months	−1.0 (CI not reported ‡)	MAD modestly improves sleepiness
Gotsopoulos, H. et al. [33]	Randomized, controlled, two-period crossover trial	73	Placebo	AHI (events/h)	1 month	−13.0 (CI not reported ‡)	MAD superior vs. sham
Ng, A. T. et al. [35]	Randomized crossover RCT	10-Oct	Placebo	AHI (events/h)	1 week	−11.8 (CI not reported ‡)	MAD superior vs. sham

Footnotes to Table 2: * Sydney crossover ESS: the paired treatment effect was reported as CPAP − MAD = 0.31 (95% CI −0.2 to 0.9) and converted to MAD − CPAP = −0.31 (95% CI −0.90 to +0.20) for consistency with table directionality. ** CHOICE trial: the reported contrast CPAP − MAD = 11.4 (95% CI 7.9 to 14.9) was converted to MAD − CPAP = −11.4 (95% CI −14.9 to −7.9). † Crossover trials require paired dispersion measures to calculate valid 95% confidence intervals (e.g., SE/SD of within-subject differences, or a reported paired treatment-effect CI). Condition-level SE/SEM values alone do not permit reconstruction of paired confidence intervals; therefore, point estimates are shown without CIs unless paired CIs were explicitly reported. ‡ Sham-controlled crossover trials reported condition-level means (±SD or ±SEM) without paired treatment-effect variance; therefore, point estimates are presented without 95% confidence intervals. Abbreviations: AHI, apnea–hypopnea index; ODI, oxygen desaturation index; ESS, Epworth Sleepiness Scale; MAD, mandibular advancement device.

Across placebo-controlled trials, MAD demonstrated clear absolute efficacy beyond placebo, reducing AHI relative to sham by approximately 6–13 events/h across follow-up intervals ranging from 1 week to 3 months, and producing modest improvements in sleepiness where reported. Collectively, these findings support MAD as an effective therapy compared with sham while reinforcing that CPAP remains more physiologically efficacious for normalizing objective OSA severity measures. Beyond improvements in AHI and symptoms, recent studies have examined whether MADs provide cardiovascular benefits similar to those of CPAP. An RCT involving 220 hypertensive patients with OSA comparing MAD and CPAP therapy over a period of 6 months revealed that MAD therapy reduced 24 h mean arterial pressure by 2.5 mmHg, versus no change with CPAP, and the between-group difference met the predefined noninferiority margin, establishing MAD as noninferior to CPAP for blood pressure reduction [39,40]. These findings suggest that, in properly selected patients, MAD therapy may not only alleviate OSA symptoms but also favorably impact cardiovascular risk factors, such as blood pressure. That said, CPAP remains more consistently effective for severe OSA or in patients with minimal mandibular protrusive capacity [11].

When adherence to therapy is accounted for, studies have shown that short-term health outcomes at one month of optimal therapy were comparable between groups [36]. Notably, MAD users also reported greater improvement in some general vitality domains of quality of life, suggesting that CPAP’s superior physiologic efficacy can be offset by its lower adherence relative to MAD, leading to similar overall treatment effectiveness in patients with mild to moderate OSA [36,41].

A central clinical challenge in obstructive sleep apnea management is balancing maximal physiologic efficacy with long-term treatment adherence, a tension most clearly illustrated by the comparison between continuous positive airway pressure (CPAP) and mandibular advancement devices (MADs) [10]. CPAP reliably produces the largest reductions in apnea–hypopnea index (AHI) across all severity strata and remains the most effective therapy for normalizing upper airway obstruction when used consistently [9]. However, long-term adherence to CPAP is frequently suboptimal, with a substantial proportion of patients using therapy for insufficient duration or discontinuing treatment altogether, thereby attenuating real-world effectiveness [9].

In contrast, MADs typically achieve smaller per-night reductions in AHI compared with CPAP, particularly in moderate-to-severe disease [36]. Despite this lower physiologic efficacy, MADs often demonstrate higher nightly use and long-term persistence, resulting in comparable improvements in patient-reported outcomes such as daytime sleepiness and quality of life in patients with mild-to-moderate OSA [24,36]. This apparent paradox: lower physiologic efficacy but similar clinical benefit, reflects the importance of adherence as a determinant of treatment effectiveness. When adherence is incorporated into outcome assessment, the cumulative therapeutic exposure of MAD therapy may approximate or exceed that of inconsistently used CPAP [42]. For many patients, particularly those with mild-to-moderate OSA, a slightly less efficacious but well-tolerated therapy may yield superior long-term outcomes compared with a highly efficacious therapy that is poorly adhered to.

**Figure 2:**
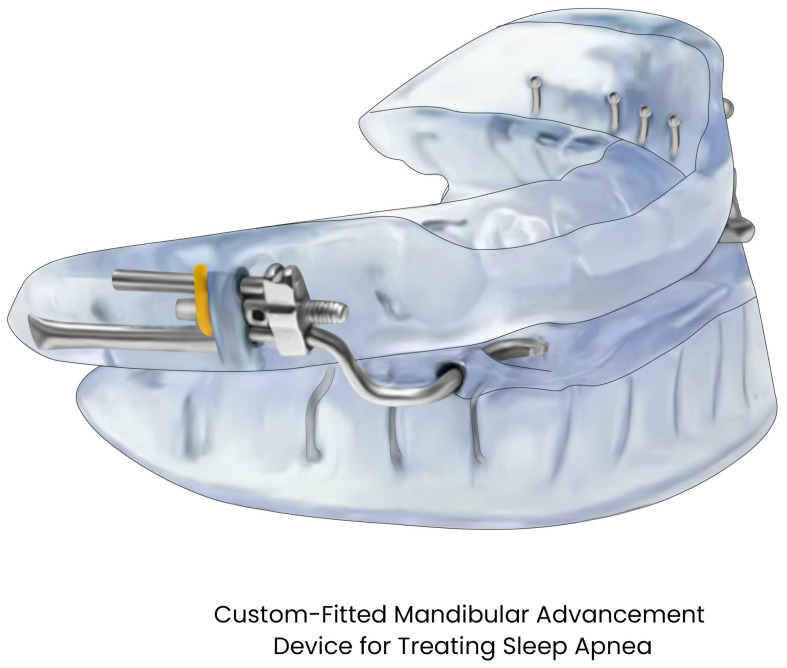
Custom-fitted, titratable mandibular advancement device for obstructive sleep apnea. Example of a custom oral appliance designed to advance the mandible during sleep. Adjustable components allow incremental titration of mandibular protrusion to optimize upper airway patency while balancing comfort and long-term tolerability.

From a precision-medicine perspective, the choice between CPAP and MAD should therefore be individualized, taking into account disease severity, anatomic predictors of MAD response, patient preference, and likelihood of sustained adherence. Phenotypic characteristics can determine the efficacy of MAD therapy to some extent, as shown by drug-induced sleep endoscopy (DISE) studies, suggesting that patients with predominantly retrolingual airway collapse respond more favorably to MAD therapy than those with retropalatal or lateral pharyngeal wall collapse [43,44]. Additionally, in clinical practice, titration is often required to optimize therapeutic benefit. Most devices are adjustable, allowing for gradual mandibular advancement until satisfactory control of OSA is achieved or jaw discomfort limits further protrusion (Figure 2).

Consistent with the AASM recommendations, efficacy should be objectively confirmed with follow-up sleep testing after titration. Across RCTs, approximately 50–70% of appropriately selected patients experience a clinically significant reduction in AHI with MAD use, while complete remission (AHI < 5 events per h) is achieved in a smaller subset, typically 40–50% of those with mild to moderate OSA, with lower success rates observed in more severe cases [11,31,45]. Emerging techniques such as remotely controlled mandibular protrusion during sleep studies allow real-time assessment of upper airway responsiveness, potentially enabling the prediction of MAD efficacy before fabrication. Additionally, combination approaches, such as MAS with positional therapy or MAD with tongue myofunctional therapy, are being explored to provide additive therapeutic benefit. Modern developments allow embedding microsensors, such as temperature sensors, within mandibular advancement devices to yield objective nightly usage data, which can provide compliance data to ensure therapeutic protrusion is maintained [46]. Concurrently, the use of intraoral scanning and computer-aided design and manufacturing (CAD/CAM) technologies has improved device fit, comfort, and reproducibility, representing a significant step toward individualized treatment of OSA [47].

The efficacy of MAD therapy is inherently variable, with some patients achieving near complete resolution of OSA and others experiencing only modest improvement [36]. Predictors of a favorable response to MAD therapy include milder baseline OSA, positional (supine-dependent) OSA, lower body mass index (BMI), and certain craniofacial characteristics, such as that can be functionally corrected by mandibular protrusion [28,36,48] (Table 3).

Primary clinical and longitudinal studies indicate that MADs are generally well tolerated, with most adverse effects being mild and reversible. Common short-term side effects include jaw or tooth discomfort, dry mouth, excessive salivation, and transient temporomandibular joint (TMJ) pain or stiffness [49]. With prolonged use, gradual dental or occlusal changes, such as reduced overjet or overbite and mesial migration of lower teeth, can occur, though these changes are typically gradual and clinically insignificant for most patients [50]. Overall, serious complications are rare, but regular dental follow-up is recommended to detect and manage occlusal changes early.

In summary, CPAP remains the preferred therapy for patients with severe OSA or high cardiometabolic risk when adherence can be achieved, whereas MADs represent a rational first-line or alternative option for patients with mild-to-moderate disease who are unlikely to tolerate CPAP. Importantly, this trade-off does not imply equivalence of therapies but rather underscores the need to distinguish physiologic efficacy from clinical effectiveness in therapeutic decision-making. The choice between CPAP and MAD should be individualized, weighing the severity of OSA, the patient’s craniofacial anatomy, and patient preference. Emerging innovations in device design, digital modeling, custom 3D-printed appliances, and built-in sensors for adherence and precision in candidate selection will likely further enhance the role of oral appliances in OSA management.

**Table 3:** Predictors of Response to Mandibular Advancement Devices. This table summarizes clinical, anatomic, and physiologic factors associated with treatment response to mandibular advancement device (MAD) therapy in adults with obstructive sleep apnea. Predictors are derived from randomized controlled trials, cohort studies, and systematic reviews evaluating objective reductions in apnea–hypopnea index and improvements in patient-reported outcomes. Identified predictors include disease severity, craniofacial morphology, upper airway anatomy, positional dependence, body mass index, and physiologic endotypes. The table is intended to guide phenotype-based patient selection rather than to define absolute contraindications [11,36,51,52,53].

Predictor	Association with MAD Response	Physiologic Rationale
Lower Body Mass Index (BMI)	Improved efficacy and higher likelihood of clinically significant AHI reduction	Reduced parapharyngeal fat and tongue volume decreases upper-airway collapsibility, allowing mandibular advancement to more effectively stabilize the pharyngeal airway
Positional (Supine-Dependent) OSA	Higher treatment success and greater AHI reduction	Mandibular protrusion counteracts posterior tongue displacement that predominates in the supine position, improving retrolingual airway patency
Retrognathia or Micrognathia	Favorable response, particularly in mild–moderate OSA	Skeletal mandibular deficiency contributes to posterior tongue positioning; anterior mandibular advancement directly corrects this anatomical predisposition
Predominant Tongue-Base (Retrolingual) Collapse	Better outcomes compared with palatal or lateral wall collapse	MADs preferentially advance the tongue and hyoid complex, making them most effective when obstruction arises primarily at the retrolingual level
Lower Baseline OSA Severity	Greater likelihood of complete or near-complete remission	Less severe airway collapsibility and ventilatory instability require smaller anatomic corrections to restore airway patency
Preserved Mandibular Protrusive Capacity	Improved tolerability and titration to therapeutic advancement	Adequate protrusive range permits optimization of airway opening without inducing temporomandibular discomfort or occlusal strain

## 4. Surgical Therapies for Obstructive Sleep Apnea

Surgical interventions for OSA are designed to remove, reposition, or modify upper airway structures to enlarge the airway lumen and reduce its tendency to collapse during sleep. In contrast to nightly therapies such as CPAP or MAD, surgical procedures can provide a fixed anatomical correction, offering long-term benefit without the need for ongoing device use. Surgical management is particularly attractive for patients who are unable or unwilling to tolerate CPAP or MAD. However, surgical success depends heavily on patient characteristics, including anatomy, BMI, and collapse sites. The major surgical advances for OSA include uvulopalatopharyngoplasty (UPPP) and variants of soft palate surgery, as well as skeletal jaw advancements such as maxillomandibular advancement (MMA) (Table 4). Neurostimulation-based techniques, particularly hypoglossal nerve stimulation (HNS), have emerged as an increasingly common and effective therapeutic modality. The following section will review these modalities in greater detail, with particular attention to evolving techniques, including unilateral versus bilateral HNS systems and targeted stimulation strategies.

Historically, tracheotomy was the first definitive and highly effective surgical treatment for severe obstructive sleep apnea, bypassing upper airway obstruction entirely and eliminating obstructive events. Early reports in the 1960s and 1970s demonstrated dramatic reversal of hypersomnolence and cardiopulmonary complications following tracheotomy in patients with severe OSA [54]. However, due to its significant lifestyle impact, stoma care requirements, and complication profile, tracheotomy is now reserved for life-threatening or refractory cases. Subsequent surgical efforts, therefore, focused on modifying upper airway anatomy, leading to the development of uvulopalatopharyngoplasty and later multilevel and skeletal advancement procedures.

**Table 4:** Surgical treatment options for adult obstructive sleep apnea. This table summarizes surgical interventions for adult obstructive sleep apnea according to anatomical target level, mechanism of action, candidate profile, expected reduction in apnea–hypopnea index (AHI), and key clinical considerations. Procedures range from isolated anatomical modifications (e.g., tonsillectomy) to multilevel skeletal advancement and neuromodulation. Adjunctive nasal surgery is included due to its role in improving positive airway pressure tolerance rather than as a definitive therapy. The table reflects adult treatment paradigms and does not address pediatric surgical management [12,17,19,23,55,56,57,58].

Procedure	Primary Target Level	Mechanism of Action	Ideal Candidate	Expected Effect on AHI	Key Considerations
Tonsillectomy (Adult)	Retropalatal/Lateral pharyngeal wall	Removal of hypertrophic palatine tonsils enlarges the retropalatal airway and reduces lateral wall collapse	Adults with significant tonsillar hypertrophy (Friedman stage I–II), mild–moderate OSA, minimal skeletal restriction	Moderate AHI reduction (approximately 20–50%) in carefully selected patients	Highly anatomy-dependent; limited benefit without enlarged tonsils; postoperative pain and short-term dysphagia
Uvulopalatopharyngoplasty (UPPP)	Retropalatal/Lateral pharyngeal wall	Resection and repositioning of soft palate and uvula reduce retropalatal obstruction	Mild–moderate OSA with palatal-dominant collapse; CPAP intolerance	Moderate AHI reduction (approximately 30–50%) with variability	Variable long-term durability; irreversible anatomical changes
Maxillomandibular Advancement (MMA)	Retropalatal + Retrolingual (Multilevel)	Skeletal advancement enlarges upper airway and increases longitudinal tension	Moderate–severe OSA with craniofacial restriction; CPAP intolerance	Large AHI reduction (approximately 60–80%); durable	Major surgery; recovery time; higher cost
Hypoglossal Nerve Stimulation (HNS)	Retrolingual/Multilevel	Inspiratory-synchronized stimulation advances and stabilizes the tongue	Moderate–severe OSA; CPAP intolerance; absence of concentric palatal collapse	Substantial AHI reduction (approximately 50–70%) in eligible patients	High upfront cost; strict eligibility criteria; limited data beyond 5 years
Nasal Surgery (Septoplasty/Turbinate Reduction)—Adjunctive	Nasal airway	Reduces nasal resistance and intranasal pressure gradients, improving airflow	Adults with significant nasal obstruction limiting CPAP tolerance	Minimal direct AHI reduction when isolated; improves CPAP tolerance and adherence	Rarely curative alone; best used to facilitate primary therapy
Tracheostomy (Salvage)	Bypasses upper airway	Creates a permanent airway distal to obstruction	Life-threatening or refractory severe OSA	Complete elimination of obstructive events	Reserved for extreme cases; significant lifestyle impact

## 5. Uvulopalatopharyngoplasty

**Figure 3:**
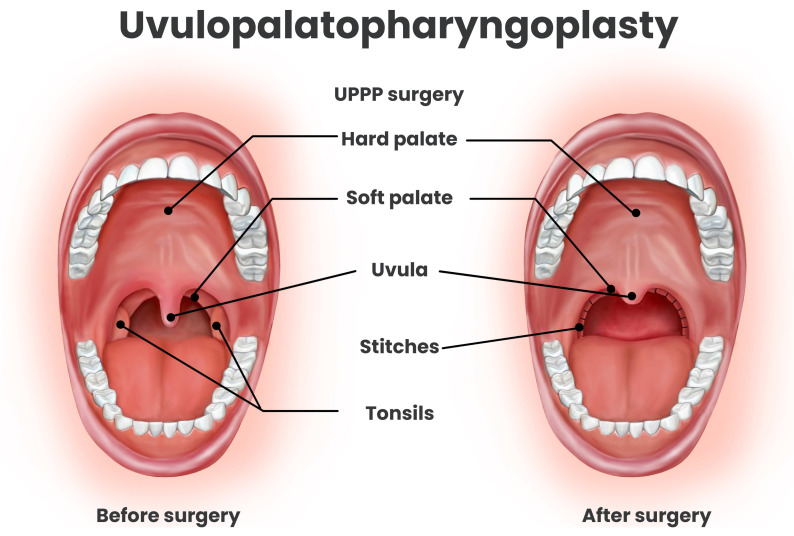
Upper airway anatomy before and after uvulopalatopharyngoplasty (UPPP). Illustration depicting the oropharyngeal anatomy before (**left**) and after (**right**) uvulopalatopharyngoplasty. Surgical resection and repositioning of the uvula and portions of the soft palate enlarge the retropalatal airway and reduce soft tissue redundancy, with the goal of decreasing palatal collapse during sleep.

Uvulopalatopharyngoplasty (UPPP) was one of the earliest surgical interventions developed for the treatment of OSA and remains the most commonly performed oropharyngeal surgery worldwide. The procedure involves resection or trimming of the uvula, a portion of the soft palate, and redundant pharyngeal tissue to enlarge the retropalatal airway and reduce upper airway collapsibility. Tonsillectomy is frequently performed concurrently with UPPP [59]. Figure 3. Numerous case series have reported success in some patients, particularly those with large tonsils or obvious soft palate elongation [58]. However, outcomes have been highly variable owing to heterogeneity in surgical technique, patient anatomy, selection criteria, and the procedure carries moderate morbidity in terms of postoperative pain, velopharyngeal dysfunction, swallowing or voice changes, and bleeding [60,61]. To enhance efficacy and minimize complications, several modifications of the original UPPP technique have been developed. Among these, expansion sphincter pharyngoplasty (ESP) and lateral pharyngoplasty aim to improve lateral pharyngeal wall tension and airway stability. ESP, in particular, has demonstrated superior outcomes compared to traditional UPPP, with cohort studies and systematic reviews reporting surgical success rates of approximately 60–80% [62,63]. These refinements represent a significant evolution in palatal surgery, emphasizing tailored anatomic correction to optimize both efficacy and safety in OSA management.

In appropriately selected patients, UPPP can lead to substantial improvement in the severity and symptoms of OSA. See Table 3 and Table 4.

Across randomized controlled trials noted in Table 5 and Table 6, UPPP-based surgery resulted in clinically meaningful short-term reductions in OSA severity compared with non-surgical comparators. In the SAMS randomized clinical trial, multilevel upper airway surgery reduced AHI by 17.6 events/h more than medical management at 6 months and was associated with a large improvement in daytime sleepiness [64]. Similarly, in a randomized controlled trial of tonsillectomy with UPPP, surgical treatment reduced AHI by approximately 11 events/h more than control at 3 months [58]. However, the long-term durability of UPPP appears limited. In prospective cohorts with extended follow-up, initial reductions in AHI at 1–2 years were not sustained, with AHI returning toward baseline by 8 years [65].

These findings, as noted in Table 5, show that UPPP provides meaningful short-term physiologic and symptomatic benefit in carefully selected patients with oropharyngeal obstruction and CPAP intolerance. Randomized trials demonstrate reductions in AHI comparable to or exceeding those seen with conservative medical management over short follow-up intervals. In contrast, durability is a major limitation. Long-term observational data consistently show attenuation or loss of benefit, suggesting that UPPP primarily modifies dynamic soft-tissue behavior rather than addressing fixed anatomic or neuromuscular contributors to airway collapse. This mechanistic limitation likely explains the inferior long-term outcomes observed relative to therapies that provide continuous pneumatic splinting (CPAP), neuromodulation (hypoglossal nerve stimulation), or skeletal expansion (maxillomandibular advancement).

Across additional case series and systematic reviews, UPPP has demonstrated surgical success in a majority of appropriately selected patients with OSA, particularly in those with favorable anatomic features such as enlarged tonsils or elongated soft palates [61,67]. These findings emphasize the importance of meticulous patient selection, often aided by preoperative drug-induced sleep endoscopy (DISE) to identify specific sites and patterns of upper airway collapse. In contrast, outcomes tend to be less favorable in patients with macroglossia, retrognathia, or obesity, as these features contribute to persistent airway obstruction at nonpalatal levels. Technique variability and inconsistent inclusion or exclusion of tonsillectomy have historically limited the generalizability of UPPP outcomes [61]. While some studies suggest that patients with BMI values approaching 40 kg/m^2^ may still benefit under specific circumstances, most surgeons remain cautious in operating on markedly obese patients, as obesity related tongue base collapse may blunt the benefit of isolated palatal surgery [68,69]. To address these limitations and expand indications, a range of UPPP variants have been developed, including the uvulopalatal flap, lateral pharyngoplasty, Z-palatoplasty, and expansion sphincter pharyngoplasty. Multilevel approaches that combine palatal surgery with tongue base reduction or nasal surgery have also been introduced to target complex, multilevel obstruction and enhance surgical consistency and outcomes [70,71,72,73,74].

Adverse effects following uvulopalatopharyngoplasty (UPPP) are generally mild and transient, though a range of potential complications has been described. Common side effects include postoperative pain, velopharyngeal insufficiency, persistent throat dryness, and changes in voice quality. More serious complications, such as bleeding or infection, are rare but can occur [75]. UPPP often results in a durable reduction in snoring; however, if residual OSA persists, the surgically altered anatomy may complicate subsequent management, and adjunctive therapies—such as CPAP, oral appliance therapy, or weight reduction—may still be required. Despite these limitations, UPPP remains a valuable therapeutic option for patients unable to tolerate CPAP or MADs, particularly when anatomical findings are favorable for palatal intervention. Patient satisfaction tends to be highest when expectations are appropriately set, and residual OSA is modest. Overall, UPPP should be performed by experienced surgeons using an individualized, anatomy-driven approach. Preoperative counseling is essential, as approximately 40–50% of patients may have residual OSA postoperatively, necessitating follow-up sleep testing and possible adjunctive therapy [76].

In summary, UPPP remains a valuable treatment option for appropriately selected patients—most commonly those with moderate OSA, evident palatal obstruction, and intolerance or poor adaptation to CPAP or MAD therapy. When performed with careful technique, UPPP can achieve significant reductions in AHI and ODI, with high patient satisfaction. Optimal outcomes depend on meticulous patient selection and surgical execution that maximizes airway enlargement while minimizing postoperative scarring. Within a comprehensive, multimodal management strategy, UPPP may also be combined with complementary procedures such as nasal septoplasty, tongue-base reduction, or subsequent hypoglossal nerve stimulation to address multi-level airway obstruction and enhance overall treatment efficacy.

## 6. Maxillomandibular Advancement

**Figure 4:**
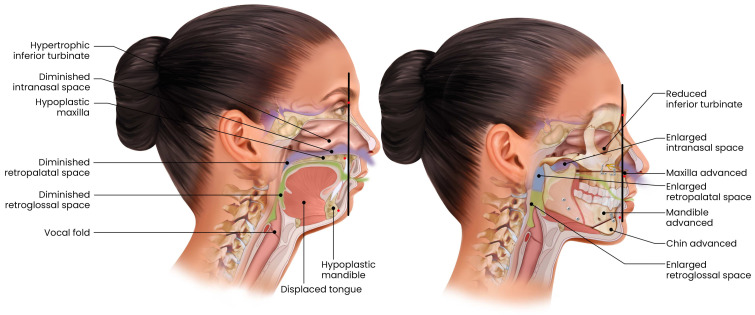
Upper airway and craniofacial anatomy before and after maxillomandibular advancement (MMA). Sagittal illustration demonstrating craniofacial and upper airway anatomy before (**left**) and after (**right**) maxillomandibular advancement. Surgical advancement of the maxilla and mandible enlarges the retropalatal and retrolingual airway spaces, advances the tongue base, and increases intranasal and pharyngeal airway volume, resulting in reduced upper airway collapsibility during sleep.

Maxillomandibular advancement (MMA) is an orthopedic surgical procedure that advances both the maxilla and mandible anteriorly, thereby enlarging the skeletal framework of the upper airway. Figure 4. This forward repositioning effectively expands the entire pharyngeal airway, from the nasal passages to the hypopharynx, by concurrently pulling the soft palate forward via maxillary advancement and the tongue base forward through mandibular advancement. As such, MMA provides a multilevel airway enlargement in a single operation. The procedure is often performed in conjunction with genial tubercle advancement and suspension of the suprahyoid muscles to further augment airway patency [56,77]. Although MMA is a major surgical intervention typically requiring intraoral incisions, rigid fixation with plates and screws, and a postoperative recovery period involving jaw stabilization, it yields the highest cure and response rates among all surgical treatments for OSA. MMA is particularly appropriate for patients with moderate to severe OSA who have underlying craniofacial skeletal deficiencies that contribute to airway restriction. Careful patient selection, based on craniofacial structure and severity of disease, is crucial to optimize surgical outcomes and minimize unnecessary intervention in those less likely to benefit [78]. MMA has demonstrated robust and durable efficacy in patients with moderate to severe obstructive sleep apnea (OSA).

Across controlled trials noted in Table 7 and Table 8, MMA produced large reductions in objective OSA severity and achieved physiologic efficacy comparable to positive airway pressure therapy. In a randomized trial comparing MMA with APAP, no statistically significant differences were observed between groups in improvement in AHI or Epworth Sleepiness Scale scores, indicating comparable short-term efficacy [79]. Similarly, in a prospective within-patient comparison, residual disease severity following MMA was nearly identical to that observed during CPAP therapy [80]. Comparative surgical data demonstrate that MMA is significantly more effective than palatal surgery alone [17]. In a baseline-adjusted cohort, MMA reduced AHI by approximately 21 events/h more than UPPP, and the addition of palatal surgery to MMA did not confer additional benefit [17]. Real-world comparative effectiveness data further show that MMA achieves the highest mean disease alleviation among CPAP, MAD, hypoglossal nerve stimulation, and MMA, reflecting adherence-independent disease control [81]. Prospective mechanistic studies indicate that MMA often results in near-normalization of AHI and high cure rates [82]. Additionally, across multiple meta-analyses, MMA has been associated with a mean reduction in AHI of approximately 77%, with nearly half of patients (47%) achieving complete remission, defined as an AHI < 5 events per h [83]. Meta-analyses have consistently confirmed these findings, reporting surgical success rates of 80–90%, usually defined as a ≥50% reduction in AHI to <20 events per h, with mean reductions in AHI often exceeding 30–40 events per h [84,85]. Long-term follow-up investigations further confirm the durability of these outcomes, demonstrating sustained improvements in AHI, oxygen saturation, and symptom relief at five years or more postoperatively [86]. In addition to ameliorating sleep-disordered breathing, MMA has been associated with improvements in cardiovascular risk factors and reductions in daytime sleepiness as measured by validated self-report instruments [87].

Collectively, these findings establish MMA as the most efficacious surgical therapy for moderate to severe obstructive sleep apnea in appropriately selected patients. Unlike soft-tissue procedures, MMA addresses fixed skeletal and tongue-base contributors to airway collapse, producing large and often durable reductions in disease severity. The absence of additional benefit from concomitant palatal surgery underscores the dominant role of skeletal advancement in achieving airway stability. Importantly, randomized and physiologic comparisons indicate that MMA can achieve disease control comparable to CPAP, while offering an adherence-independent treatment option. Real-world effectiveness analyses further support MMA’s clinical impact, demonstrating superior mean disease alleviation relative to non-surgical therapies and other surgical modalities. These advantages must be balanced against the invasiveness of the procedure and the need for careful patient selection, but for patients with severe OSA, craniofacial restriction, or CPAP intolerance, MMA represents a definitive and durable therapeutic strategy.

MMA is commonly performed by oral and maxillofacial surgeons and often in collaboration with orthodontists when preoperative dental alignment or postoperative occlusal adjustments are required. The procedure is conducted under general anesthesia and generally necessitates a brief hospital stay. Rigid fixation of the osteotomized segments is achieved using titanium plates and screws; with modern techniques, stable fixation can be accomplished without prolonged maxillomandibular wiring, although a soft diet is recommended for several weeks during recovery. Postoperative swelling and discomfort are expected and may be significant in the early period. Potential surgical risks include infection, malocclusion, and transient or, less commonly, persistent sensory changes. Because the jaws are advanced anteriorly, most patients experience some degree of facial alteration, which should be thoroughly discussed during preoperative counseling. Despite its invasiveness, serious complications from MMA are relatively uncommon. Permanent sensory nerve deficits may occur, but are generally mild, and minor occlusal discrepancies can often be corrected with secondary orthodontic adjustments or limited revision procedures.

Because MMA simultaneously addresses multiple levels of airway obstruction through skeletal repositioning, it is generally reserved for patients with moderate to severe OSA, particularly those with identifiable craniofacial abnormalities such as maxillary or mandibular retrusion, or as a salvage option following unsuccessful soft-tissue surgeries Current clinical guidelines typically recommend MMA for severe or refractory cases due to its complexity; however, it may also serve as a primary surgical intervention in patients with favorable craniofacial anatomy [12]. By advancing both the maxilla and mandible, MMA corrects multisegmental airway collapse without removing soft tissue, thereby preserving the option for adjunctive therapies if needed postoperatively. In addition to its anatomic benefits, MMA has been shown to improve OSA-related outcomes such as hypertension and daytime sleepiness to a degree comparable to, or even exceeding, that achieved with CPAP [83]. However, not all patients are ideal candidates. The procedure is most effective in individuals with craniofacial skeletal deficiencies or occlusal abnormalities that can be simultaneously corrected. In contrast, patients with normal jaw structure and obesity-related airway narrowing may derive less benefit, as excess soft tissue and tongue fat remain potential sources of obstruction. For such patients, weight reduction and metabolic optimization remain important complementary strategies to enhance overall treatment success.

In summary, MMA represents one of the most effective and durable surgical treatments for OSA, particularly in patients with craniofacial skeletal deficiencies or severe disease who are CPAP intolerant. The procedure achieves substantial reductions in AHI and high cure rates, accompanied by meaningful improvements in blood pressure, daytime symptoms, and overall quality of life. Although MMA is a major surgical intervention requiring specialized expertise, complication rates are relatively low, and patient satisfaction is high when appropriately selected and executed. MMA should be considered an integral part of the therapeutic discussion, especially in those with anatomic phenotypes conducive to skeletal advancement.

## 7. Hypoglossal Nerve Stimulation

**Figure 5:**
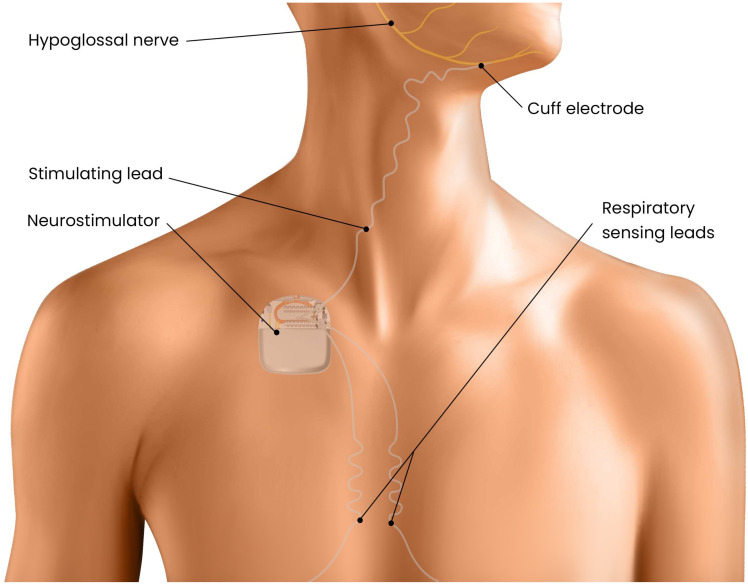
Components and mechanism of hypoglossal nerve stimulation (HNS) therapy for obstructive sleep apnea. Illustration of an implantable unilateral hypoglossal nerve stimulation system showing the neurostimulator, stimulating lead with cuff electrode placed on the hypoglossal nerve, and respiratory sensing leads. The system detects inspiratory effort and delivers synchronized stimulation to advance and stabilize the tongue during sleep, reducing upper airway collapse.

**Figure 6:**
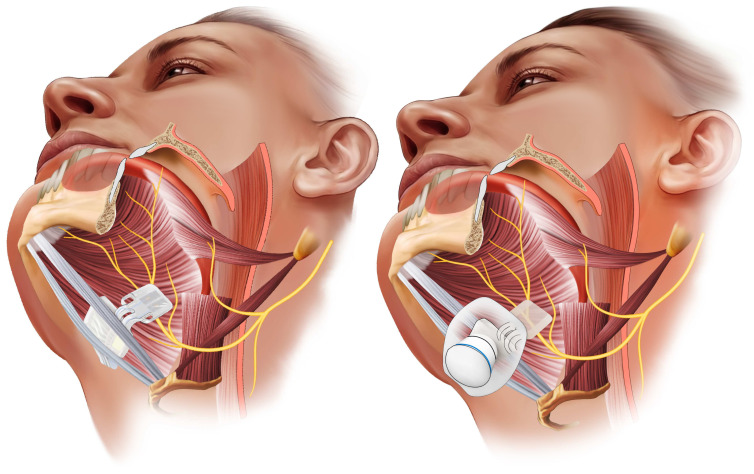
Bilateral hypoglossal nerve stimulation. Unlike earlier implantable hypoglossal nerve stimulation devices that require tunneled leads connecting a pulse generator to a cuff electrode and respiratory sensing lead, this system utilizes a small, battery-free submental stimulator implanted through a single incision without tunneling. The device delivers bilateral stimulation to the hypoglossal nerve branches via implanted electrodes and is activated by an externally worn unit that transmits energy transcutaneously.

**Figure 7:**
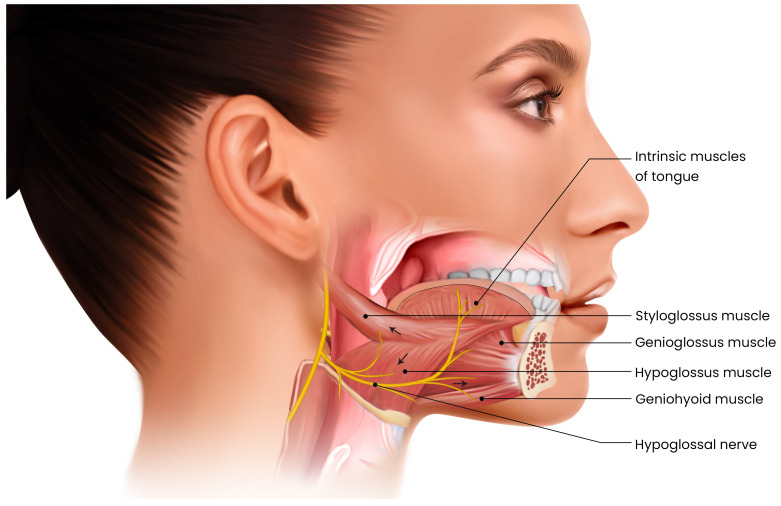
Hypoglossal nerve innervation of tongue musculature targeted by hypoglossal nerve stimulation. Sagittal illustration showing the hypoglossal nerve and its innervation of the genioglossus and other intrinsic and extrinsic tongue muscles involved in tongue protrusion and upper airway stiffening. Activation of these muscles during sleep advances and stabilizes the tongue, reducing pharyngeal collapse in obstructive sleep apnea.

Hypoglossal nerve stimulation (HNS) is a relatively new surgical therapy for OSA that involves implanting a neurostimulator to electrically activate the hypoglossal nerve during sleep. The hypoglossal nerve innervates the genioglossus and other tongue muscles responsible for tongue protrusion and upper airway stiffening. Figure 5, Figure 6 and Figure 7. By delivering synchronized stimulation with each inspiration, HNS promotes forward tongue movement and stabilization, thereby preventing upper airway collapse without the need for an external mask or positive airway pressure [19]. Over the past decade, HNS has emerged as a promising alternative for patients with moderate to severe OSA who are unable to tolerate CPAP. The therapy received U.S. Food and Drug Administration (FDA) approval in 2014 following the pivotal STAR trial, and several HNS systems have since been developed and evaluated. Current indications generally include patients with moderate to severe OSA, a BMI below 32 kg/m^2^, and the absence of complete concentric palatal collapse as determined by drug-induced sleep endoscopy [88]. Recent technological advances include bilateral stimulation systems, refined electrode configurations, and targeted activation of specific hypoglossal nerve branches to enhance efficacy and comfort.

The unilateral HNS system, the most widely used device in the United States, comprises three implanted components placed under general anesthesia. The first is a stimulation lead that encircles the hypoglossal nerve, usually in the medial branches, which are responsible for tongue protrusion, on one side of the neck. The second is a sensing lead positioned between the intercostal muscles to detect respiratory effort and timing. The third component is an implantable pulse generator, similar in design to a cardiac pacemaker, which is positioned subcutaneously in the chest. This generator receives input from the respiratory sensor and delivers synchronized stimulation to the hypoglossal nerve during each inspiratory phase [19]. The system functions by activating the genioglossus and related muscles during inspiration, thereby advancing the tongue forward to maintain airway patency, and ceasing stimulation during expiration. Following implantation, the device is calibrated during a postoperative polysomnography to optimize stimulation amplitude and timing, maximizing airway opening while minimizing arousals or discomfort. Patients control the device externally, turning it on before sleep and off upon awakening, with a built-in delay that allows sufficient time to fall asleep comfortably.

Across prospective trials (Table 9), long-term follow-up studies, and real-world registries, hypoglossal nerve stimulation was associated with large and sustained reductions in obstructive sleep apnea severity. In the pivotal STAR cohort, median AHI decreased by 68% at 12 months, accompanied by parallel reductions in ODI and normalization of daytime sleepiness. These improvements were durable, with a 5-year follow-up demonstrating sustained reductions in AHI (mean change approximately −20 events/h) and stable responder rates between 63–75%, even under conservative missing-data assumptions. Overall, 66% of participants met responder criteria, defined as a ≥50% reduction in AHI and a post-treatment AHI < 20 events per h. In a randomized therapy withdrawal sub-study, temporary cessation of stimulation led to the recurrence of OSA severity and associated symptoms, confirming that the observed benefits were directly attributable to active neurostimulation. Serious adverse events occurred in fewer than 2% of subjects. The most common side effects included tongue soreness, mild incisional discomfort, and transient tongue weakness, and were typically mild and self-limited. These findings strongly establish unilateral HNS as an effective, durable, and well-tolerated therapy for appropriately selected CPAP-intolerant patients with OSA.

Long-term follow-up of hypoglossal nerve stimulation (HNS) supports sustained therapeutic effect, adherence, and safety in appropriately selected OSA patients [89]. At five years, participants from the STAR cohort continued to exhibit significant reductions in AHI, along with persistent improvements in daytime sleepiness, as measured by the ESS, and in quality-of-life assessments, without evidence of increased stimulation thresholds or notable tongue-related injury [89]. Similarly, data from the ADHERE registry have confirmed strong real-world adherence, with average nightly device use of approximately 5.6 h, high patient satisfaction, and durable improvements in objective sleep parameters through 12 months of follow-up [90]. Device durability is also favorable; battery replacement analyses indicate that implantable pulse generators typically last approximately 10 years before requiring replacement, reflecting strong long-term reliability [91]. Although HNS carries a considerable initial cost, health economic models suggest it is cost-effective in CPAP-intolerant populations, given the substantial improvements in sleep quality, daytime function, and overall quality-adjusted life years gained [92]. However, patient candidacy remains limited by anatomic and phenotypic factors. Individuals with severe obesity, complete concentric palatal collapse, or neuromuscular tongue weakness are generally poor candidates, underscoring that HNS serves as a selectively applicable alternative CPAP therapy in well-defined patient populations.

Not all patients with OSA are eligible for HNS. The optimal candidacy is limited to those with moderate to severe OSA who are CPAP intolerant, have an upper airway collapse pattern primarily amenable to tongue protrusion rather than circumferential palatal collapse, and are not markedly obese. In the STAR trial, the device’s enrollment criteria restricted BMI to ≤32 kg/m^2^ and excluded patients who exhibited complete concentric collapse (CCC) at the soft palate on DISE, based on early evidence that CCC undermines the response to tongue-based stimulation [19]. Subsequent observational and predictive modeling analyses consistently reinforced CCC as a major exclusion criterion, highlighting the critical role of DISE in preoperative patient selection [93]. While more recent data suggest that the candidate pool for HNS may be gradually expanding, most clinical investigations continue to exclude patients with very high AHI values or severe obesity, recognizing that, in such cases, the degree of soft-tissue loading and airway collapsibility may exceed the compensatory capacity of neurostimulation. Thus, HNS remains a highly effective yet selectively applicable therapy, best reserved for carefully selected patients whose anatomical and physiological profiles predict a favorable response to tongue-protrusive stimulation. Recent innovations in HNS have sought to simplify implantation and broaden patient eligibility beyond the traditional system utilized in the STAR trial, which requires an implanted pulse generator and intercostal sensing lead.

One of the most promising developments is a bilateral HNS device that eliminates the need for an implanted battery or chest leads. Compared with earlier hypoglossal nerve stimulation systems, this device offers several structural and procedural advantages. It eliminates the need for implanted leads connecting sensing electrodes to a pulse generator and requires only a single surgical incision without subcutaneous tunneling [94]. In addition, stimulation is delivered to both hypoglossal nerves simultaneously and is regulated by an externally worn controller [94]. This controller activates a compact, battery-free stimulator implanted in the submental region, delivering therapy at a preset but adjustable frequency and duty cycle. The BLAST OSA trial (Table 9) followed 27 adults with moderate-to-severe OSA who were CPAP-intolerant. After 6 months of therapy, the mean AHI decreased by about 54%, and the ODI decreased from 23.7 to 12.9 events per h [94]. Patients also reported significant improvement in daytime sleepiness, with a significant decrease in ESS and improvements in sleep-specific quality of life, as measured by the Functional Outcomes of Sleep Questionnaire (FOSQ) [94]. No serious device-related adverse events occurred, and the few complications observed, such as minor incision discomfort or tongue tingling during early titration, resolved spontaneously or with adjustment of stimulation parameters. From a usability perspective, adherence to HNS therapy has been excellent, with the majority of participants using the system consistently by activating the external controller before sleep and deactivating it upon awakening. Notably, efficacy outcomes from the BLAST OSA trial are comparable to those achieved with the unilateral implanted HNS system, despite the bilateral device’s markedly simplified design. Should long-term durability data and broader clinical trials confirm these findings, bilateral, battery-free stimulation systems have the potential to substantially expand access to neurostimulation therapy for obstructive sleep apnea while simultaneously reducing surgical complexity, procedural risk, and long-term maintenance requirements.

**Table 9:** Prospective, durability, and real-world evidence describing the magnitude and persistence of benefit following hypoglossal nerve stimulation. Effect estimates are reported as within-patient changes from baseline, including absolute and relative reductions in apnea–hypopnea index (AHI), oxygen desaturation index (ODI), and Epworth Sleepiness Scale (ESS), as well as responder proportions and adherence metrics where available [19,89,90,94].

Study	Design	N	Comparator	Outcome	Time Point	Baseline	Follow-Up	Effect Size (Change)	Interpretation
Strollo et al., 2014 (STAR) [19]	Prospective cohort	126	Baseline	AHI (median)	12 months	29.3	9	−68%	Causality supported by withdrawal phase
Strollo et al., 2014 (STAR) [19]			Baseline	AHI responder rate	12 months	—	—	66%	≥50% decrease and AHI < 20
Woodson et al., 2018 [89]	Prospective durability	71	Baseline	AHI (mean)	60 months	32	12.4	−19.6 events/h	Durable for 5 years
Woodson et al., 2018 [89]			Baseline	ESS (mean)	60 months	11.6	6.9	−4.7	Sustained symptom benefit
Woodson et al., 2018 [89]			Baseline	AHI responder rate	60 months	—	—	75% (PSG cohort)	63% with LOCF
Thaler et al., 2020 (ADHERE) [90]	Multicenter registry	1017	Baseline	AHI (median)	12 months	32.8	9.5	−23.3 events/h	Real-world effectiveness
Thaler et al., 2020 (ADHERE) [90]			Baseline	ESS (mean)	12 months	11.4	7.2	−4.2	—
Eastwood et al., 2020 (BLAST OSA) [94]	Prospective single-arm	27	Baseline	AHI (mean)	6 months	23.7	12.9	−10.8 events/h	Bilateral system effective
Eastwood et al., 2020 (BLAST OSA) [94]			Baseline	ESS (mean)	6 months	11	8	−3.0	—

Footnotes: Effect estimates represent within-group change from baseline unless otherwise specified. The STAR trial included a randomized therapy-withdrawal phase supporting causal inference but did not include a concurrent sham arm. Five-year outcomes were supported by LOCF, multiple-imputation, and best-/worst-case sensitivity analyses to address missing polysomnography data. Registry data reflect real-world effectiveness and should not be interpreted as causal comparisons.

A more recent HNS technology has been evaluated in the THN3 trial, which investigated proximal targeted hypoglossal neurostimulation (THN), a system that stimulates the hypoglossal nerve more proximally using a multiconduct cuff electrode to deliver asynchronous stimulation. Mechanistically, this design allows co-activation of both tongue protrudor and retrudor intrinsic muscle groups, producing overall stiffening and stabilization of the tongue rather than solely anterior displacement. This dual muscle recruitment strategy may enhance airway patency even in anatomically complex or multilevel collapse scenarios.

In the THN3 trial, 138 patients with moderate to severe OSA who were CPAP intolerant were randomized 2:1 to early device activation versus delayed start (Table 10). At four months, 52.3% of patients in the active treatment group met the responder criterion, which was defined as a ≥50% reduction in AHI and a post-treatment AHI ≤ 20 events per hour, compared with 19.6% in the control arm, confirming the efficacy of stimulation. Over 11 months of therapy, 42.5% of the pooled cohort met the AHI responder definition, and 60.4% achieved the corresponding ODI criteria. Significant improvements were also observed across multiple patient-reported outcomes, including daytime sleepiness, sleep-related quality of life, and overall health utility [95]. Longitudinal follow-up extending to 36 months demonstrated that improvements in AHI, ODI, ESS, and quality-of-life metrics were largely sustained, with a low incidence of serious device-related complications and predominantly mild adverse effects, such as transient tongue discomfort or stimulation-related paresthesia [96]. These effects were statistically robust (Table 10), with confidence intervals excluding the null for both AHI and ODI responder outcomes. Longer-term pooled analyses demonstrated sustained responder proportions at 12–15 months, supporting persistence of benefit beyond the randomized comparison period.

In summary, HNS provides robust and durable within-patient improvement in both objective respiratory indices and patient-reported outcomes. Unlike soft-tissue surgical procedures, the durability of benefit through five years suggests that neuromodulatory stabilization of upper airway tone produces a persistent physiologic effect rather than transient symptom relief. At the same time, residual AHI following HNS typically remains higher than that observed with CPAP under optimal adherence or with maxillomandibular advancement, positioning HNS as a moderately efficacious but durable therapy. The consistency of effect across controlled trials, long-term follow-up, and large registries underscores the external validity of HNS outcomes. Importantly, the magnitude of benefit appears phenotype-dependent, with baseline physiological characteristics such as lower ODI and lower BMI predicting long-term response. These findings reinforce a precision-medicine framework, in which HNS is most appropriately deployed in carefully selected CPAP-intolerant patients rather than as a universal alternative.

## 8. Neuromuscular Electrical Stimulation Devices

Another emerging approach to treating OSA involves neuromuscular electrical stimulation (NMES) of the upper airway musculature. In contrast, HNS, which requires implanted electrodes on the nerve, these therapies stimulate the muscles more peripherally, either via intraoral or transcutaneous surface electrodes. The therapeutic objective is to enhance baseline muscle tone and responsiveness of these airway-stabilizing muscles, thereby reducing airway collapsibility during sleep. Two distinct strategies have been investigated: (1) daytime training stimulation in which electrical impulses are applied while the patient is awake to strengthen upper-airway muscles over time, and (2) nighttime stimulation, which delivers rhythmic electrical pulses during sleep to maintain airway patency dynamically. The following sections review major developments in this field, including evidence from clinical trials of daytime intraoral stimulation devices and emerging data from nocturnal transcutaneous stimulation systems currently under investigation.

### 8.1. Daytime Intraoral NMES

**Figure 8:**
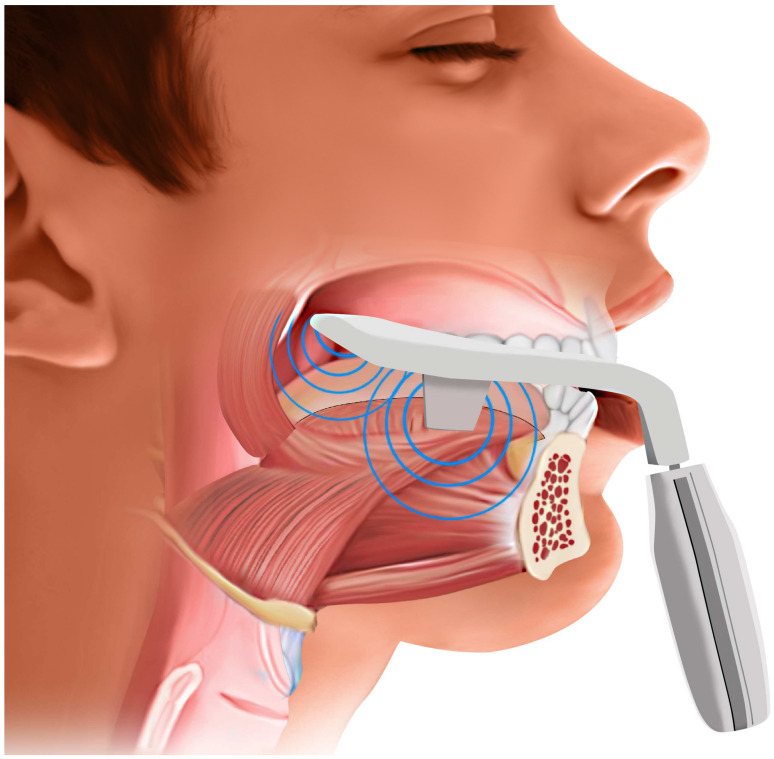
Intraoral neuromuscular electrical stimulation of tongue musculature in obstructive sleep apnea. Noninvasive intraoral neuromuscular electrical stimulation (NMES) device positioned along the sublingual region to activate intrinsic and extrinsic tongue muscles. Electrical stimulation (depicted by the blue concentric circles) targets upper airway dilator muscles, including the genioglossus, to enhance muscle tone and reduce airway collapsibility.

Recent intraoral devices have been FDA-authorized tongue muscle stimulation devices for OSA. It typically consists of a silicone mouthpiece with four electrodes, one above and one below the tongue, connected to a handheld controller and a smartphone app. The device can deliver a series of electrical pulse bursts to the tongue for a set training session, typically 20 min, while the patient is awake. Figure 8. By providing neuromuscular electrical stimulation to the tongue over a typical 6–8-week training period of daily use, this regimen aims to strengthen the tongue and improve its resting tone [97]. The concept is akin to physiotherapy for the tongue. By increasing muscle responsiveness and reducing fatty infiltration in the tongue, the device seeks to prevent the tongue from collapsing backward during sleep.

In a randomized, double-masked, sham-controlled trial involving patients with mild obstructive sleep apnea, active neuromuscular electrical stimulation resulted in a statistically significant reduction in respiratory event burden compared with sham therapy (Table 11). Active NMES produced a 32.7% reduction in respiratory event index (REI) over 6 weeks, whereas no significant change was observed in the sham group. Improvements were more pronounced in supine REI, suggesting a position-dependent neuromuscular effect on upper airway patency.

Active NMES was also associated with improvements in daytime sleepiness, as measured by the Epworth Sleepiness Scale, whereas sham therapy produced no significant symptomatic benefit. Importantly, adherence exceeded 90% in both active and sham arms, indicating excellent tolerability and feasibility of the intervention. No device-related serious adverse events were reported during the study period.

The findings from this randomized, sham-controlled trial indicate that neuromuscular electrical stimulation confers modest but clinically meaningful short-term benefits in patients with mild obstructive sleep apnea. The magnitude of REI reduction (~3–5 events/h) is substantially smaller than that reported for hypoglossal nerve stimulation or maxillomandibular advancement, but the observed effect exceeds that expected from placebo alone and is accompanied by parallel improvements in subjective sleepiness.

The position-dependent nature of response, with greater improvement in supine REI, supports a neuromuscular conditioning mechanism targeting upper airway dilator function rather than real-time airway stabilization. This mechanistic distinction differentiates NMES from implanted hypoglossal nerve stimulation, which provides synchronous nocturnal activation, and from skeletal advancement procedures that permanently modify airway anatomy.

From a clinical perspective, NMES occupies a distinct role as an adjunctive or early-stage therapy, particularly for patients with mild disease, positional OSA, or residual symptoms following other interventions. The exceptionally high adherence observed in both active and sham arms highlights the acceptability of the modality and suggests potential value in populations where adherence to PAP or oral appliances is problematic.

However, the evidence base for NMES remains limited by short follow-up duration, small sample size, and restriction to mild OSA populations. There are currently no data supporting its use as a definitive therapy for moderate-to-severe disease, nor is there evidence of long-term durability comparable to HNS or MMA. Accordingly, NMES should be viewed as complementary rather than competitive with established non-CPAP therapies.

NMES is intended for mild OSA or simple snoring. It offers an attractive option for those averse to wearing any device at night. Increasing muscle endurance and tone may mitigate mild collapsibility. It is not indicated for moderate to severe OSA as sole therapy, as its efficacy in those ranges is unproven. Also, it requires daily commitment for several weeks to see benefits, and possibly ongoing maintenance sessions a few times per week to sustain muscle conditioning. Patient acceptance has been good in studies, likely because the therapy is relatively easy to tolerate, with only slight tongue tingling during the 20 min sessions, and can be done at a convenient time. Within a precision-treatment framework, NMES may serve as a low-risk neuromuscular intervention that enhances upper airway function in carefully selected patients, while more invasive or durable therapies are reserved for those with greater disease severity or anatomic contributors to collapse.

### 8.2. Nocturnal Transcutaneous Electrical Stimulation

Another approach is to apply electrical stimulation during sleep to maintain airway patency in real time. This typically involves electrodes on the skin overlying upper airway muscles, the submental area, or the lateral neck that deliver rhythmic stimulation throughout the night. The concept dates back decades, but only recently have controlled trials evaluated modern transcutaneous electrical stimulation (TES) devices.

A randomized trial investigated whether TES of upper airway dilator muscles could acutely mitigate obstructive events in patients with OSA [98]. In a randomized, crossover study, 36 participants underwent two overnight in-laboratory polysomnography sessions, one with active TES and the other with sham stimulation, separated by a washout period. Electrical pulses were delivered via adhesive submental surface electrodes targeting the genioglossus and adjacent suprahyoid muscles, providing continuous stimulation throughout sleep. Active TES produced a statistically significant, albeit modest, improvement in sleep-disordered breathing indices. The median ODI_4_ decreased from 27 events per h during sham to 20 events per h with active stimulation, representing an average reduction of approximately 4 events per h. Similarly, the median AHI improved from 28 to 24 events per h, corresponding to an overall 15–20% reduction in event frequency across the cohort.

Notably, nearly half of the participants met predefined responder criteria, defined as either a >25% reduction in ODI_4_ or achievement of an ODI_4_ < 5 events per h during active stimulation. Logistic regression analyses identified baseline OSA severity as a key predictor of treatment response, with the greatest benefit observed among individuals with AHI < 30 events per h and lower arousal indices. These findings suggest that patients with milder airway collapsibility or reduced arousal propensity may experience the most pronounced airway stabilization from TES therapy.

The longer efficacy of TES devices has also been studied in a nightly home-based transcutaneous electrical stimulation protocol using a standard TENS device applied submentally. In a prospective trial involving 56 CPAP-intolerant patients with moderate OSA, three months of active TES therapy resulted in a median AHI reduction from 25 to 13 events per h, indicating a clinically meaningful improvement in airway stability and sleep-disordered breathing severity [99]. Daytime ESS improved significantly in the stimulation group, but not controls, and measures of sleep fragmentation also trended toward improvement with TES use [99]. Adherence was good, with a median nightly use of approximately 5 h [99]. Safety was favorable, with only minor adverse events, such as mild skin irritation or transient discomfort, being reported, with no serious events [99]. Despite not achieving robust statistical significance in adjusted AHI change, the trial provides important proof that domiciliary, non-invasive electrical stimulation of the upper airway is feasible, safe, and capable of yielding clinically meaningful improvements in OSA severity and symptoms in CPAP-intolerant patients.

Nocturnal transcutaneous NMES aims to replicate the physiological effects of HNS through a completely noninvasive approach. The principal challenges lie in delivering sufficient current to activate target muscles through the skin, without causing discomfort, and in achieving precise synchronization with respiration. Current systems generally employ preprogrammed duty cycles rather than real-time respiratory sensing, which may limit their physiological optimization. However, the observation that many participants tolerated continuous overnight stimulation is encouraging and suggests feasibility for broader clinical use. Therapeutic response appears most favorable in individuals with lower arousal propensity or milder disease severity, highlighting the importance of phenotypic screening to identify suitable candidates. Notably, pharmacologic modulation, such as the use of sedative hypnotics like eszopiclone to raise the arousal threshold, could theoretically enhance tolerance and efficacy in this subgroup. Future studies integrating such combination strategies are warranted to evaluate their synergistic potential and to further establish the safety, durability, and optimal implementation of transcutaneous NMES in OSA management.

In summary, NMES therapies have emerged as promising additions to the therapeutic landscape for OSA (Table 12). Daytime intraoral NMES has been shown to strengthen upper airway musculature over several weeks, leading to significant reductions in snoring and improvements in mild OSA in controlled trials. Nighttime transcutaneous stimulation can acutely reduce the AHI in nearly half of treated patients, particularly those with mild to moderate disease, and regular use has been associated with meaningful symptomatic benefits. These approaches are generally safe, well-tolerated, and offer a noninvasive alternative for patients who are unable or unwilling to use CPAP or undergo surgical intervention. While their efficacy in moderate to severe OSA remains below that of CPAP, NMES therapies occupy an important niche for CPAP-intolerant individuals seeking less invasive management options. Ongoing advances in device design, stimulation algorithms, and identification of responder phenotypes are expected to further improve outcomes. Moreover, combining NMES with complementary modalities, such as positional therapy, pharmacologic agents that raise the arousal threshold, or targeted behavioral interventions, may represent a future personalized, multimodal approach to OSA management.

## 9. Novel and Adjunct Techniques: Tongue Base Cryotherapy, Positional Therapy, and Other Minimally Invasive Approaches

Beyond the mainstream therapies discussed previously, several innovative techniques have recently emerged that target specific physiological mechanisms underlying OSA. These include experimental and device-based approaches, such as tongue base cryotherapy to reduce tongue volume, advanced positional therapy devices designed to discourage supine sleep, and various adjunctive measures, including nasal expiratory valves and structured myofunctional exercises to enhance upper airway tone. While each of these strategies may be most appropriate for patients with mild disease or as complementary interventions alongside other treatments, together they exemplify the expanding toolkit available for individualized, mechanism-based OSA management. The following section highlights several of these emerging therapies and reviews the current evidence supporting their clinical use.

### 9.1. Tongue Base Cryotherapy (“Tongue Freezing”)

Excess volume or laxity of the tongue base is a major contributor to airway occlusion in many OSA patients, particularly those with larger tongues or obesity-related fat deposition in the tongue. Traditional approaches to address this have included radiofrequency ablation (RFA) of the tongue base, which uses heat to create fibrosis and volume reduction, thereby stiffening and shrinking the tongue over time. RFA has shown modest efficacy. A randomized trial of temperature-controlled radiofrequency to the tongue and palate in mild to moderate OSA demonstrated significant improvements in airway volume and arousal index compared with sham, with mild, transient side effects and no serious complications [103]. However, RFA can cause postoperative pain and swelling.

**Figure 9:**
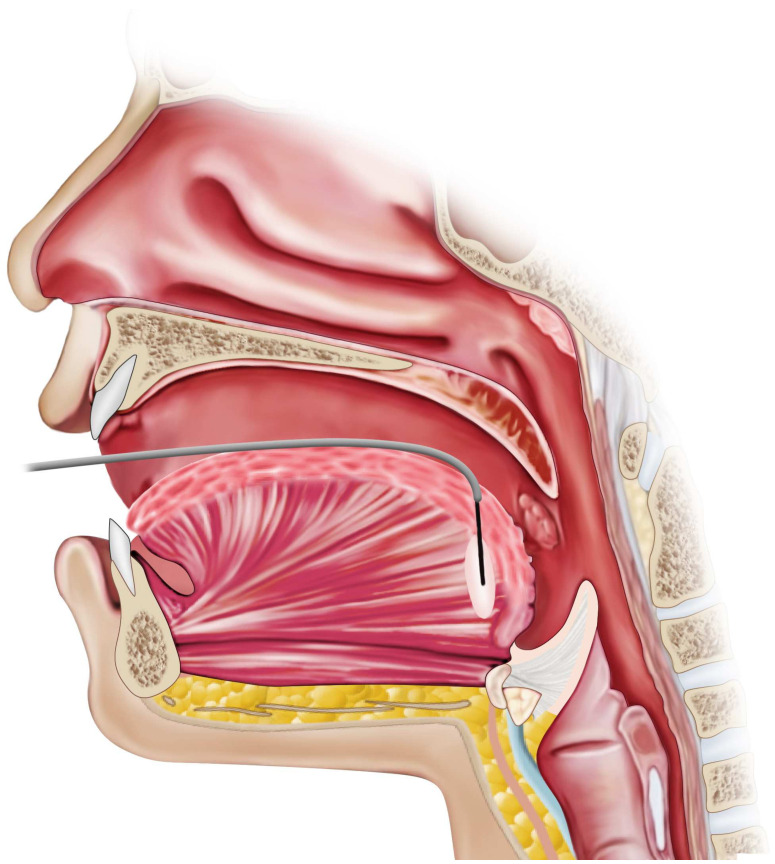
Tongue base cryotherapy for obstructive sleep apnea. Illustration of transoral tongue base cryotherapy targeting hypertrophic lingual tissue in the retrolingual airway. A cryoprobe is introduced through the oral cavity to deliver controlled cold-induced tissue ablation within the posterior tongue musculature. Subsequent tissue remodeling and volume reduction aim to decrease retrolingual airway obstruction and improve upper airway patency during sleep.

Cryotherapy is a novel approach that applies cold energy to ablate tissue, rather than heat. It involves using a probe, typically cooled by liquid nitrogen, to create controlled ice lesions in the tongue base (Figure 9). The cold causes local tissue destruction and subsequent scarring/volume reduction, but it also has an inherent analgesic effect by numbing nerves, potentially leading to less pain than RFA. Preliminary case series have reported that office-based tongue cryotherapy is feasible and could produce tongue volume reduction and AHI improvements on par with RFA, but with patients reporting lower pain scores. While robust RCT data on tongue cryoablation are not yet published, the technique has shown promise as a minimally invasive option.

Although formal peer-reviewed trials have not yet been available, a clinical development program for a cryotherapy-based tongue and soft palate ablation system, known as the ARCTIC-series, is currently underway. According to the disclosures and clinical trial registries, the ARCTIC-3 trial is a multicenter, open-label study evaluating the safety and efficacy of the Cryosa system in patients with moderate to severe OSA. Press materials indicate the first U.S. patient was treated in early 2024. The Cryosa approach utilizes an incisionless, image-guided cryoablation technique intended to reduce the volume of tongue and soft palate tissue while minimizing postoperative pain compared with conventional thermal ablative procedures. Preliminary feasibility reports suggest that treatments are performed under local anesthesia using short freeze cycles, with adverse events to date limited to mild, transient postoperative discomfort. However, in the absence of peer-reviewed publications, the magnitude of therapeutic benefit, responder rates, and long-term durability of tongue base cryotherapy remain speculative and require confirmation through rigorous, controlled human trials.

At present, tongue cryotherapy should still be considered investigational. Its role will be better defined after controlled studies measure outcomes such as AHI reduction and patient comfort relative to established tongue-reduction methods.

### 9.2. Positional Therapy Innovations

Positional OSA (POSA) is a common OSA subtype, with respiratory events occurring mainly or more frequently in the supine position than in lateral sleep. Many OSA patients meet criteria for POSA, usually defined by a supine AHI at least twice the non-supine AHI [104]. For these individuals, avoiding the supine sleeping posture can greatly reduce OSA severity. Traditional positional therapies, such as sewing a tennis ball into sleepwear or using bulky positioning belts, have been limited by discomfort, poor tolerance, and low adherence. Recently, modern vibratory positional therapy (PT) devices have been developed to address these issues. These compact, wearable sensors are typically worn on the chest or neck. They detect when the user turns to the supine position and trigger gentle vibrations that prompt a return to lateral sleep. Over time, this method aims to retrain sleep posture habits. Compared to earlier aids, modern PT devices offer much greater comfort and convenience.

Modern PT devices reliably limit back sleeping and improve objective OSA metrics in patients with positional OSA. In a randomized trial, a neck-worn vibratory device was compared with CPAP for 8 weeks each, showing that PT substantially reduced supine sleep and lowered AHI from baseline [105]. Complementary single-arm and real-world cohorts of neck-worn vibrotactile PT report good adherence and robust reductions in time supine, with significant reductions in AHI in subjects with positional OSA, with two-thirds of patients achieving ≥ 50% AHI reduction, alongside better subjective sleep quality [106]. A recent meta-analysis further confirms that vibrotactile PT reduces AHI and supine exposure in POSA, although CPAP remains superior for normalizing AHI and improving oxygenation and daytime sleepiness [107]. Collectively, these studies indicate that positional devices work as intended, reducing supine time and AHI, and can be a useful option for adherent POSA patients. However, it is important to recognize that while modern PT devices can substantially reduce AHI and improve symptoms in appropriately selected patients, they rarely achieve the degree of AHI normalization or symptom resolution observed with CPAP, which remains the reference standard when tolerated [105].

For patients with POSA who are CPAP intolerant, modern vibratory PT devices represent an excellent alternative. These compact systems are easy to use, highly portable, and generally well tolerated, with minimal side effects aside from occasional mild skin irritation or transient sleep disturbance during the initial adaptation period. Over time, most users habituate to the gentle vibratory cues and subconsciously avoid the supine position without awakening. The cost of PT devices is modest compared with CPAP or HNS therapies, further supporting their practical appeal. Because positional devices do not mechanically enlarge or splint the airway but instead prevent exposure to the most collapse-prone sleep posture, their effectiveness is greatest in patients with mild-to-moderate OSA who sleep laterally. In such cases, consistent avoidance of the supine position may normalize the AHI or reduce it to the mild range, effectively controlling disease severity. Conversely, for patients who continue to experience significant respiratory events even in the lateral position, PT alone is insufficient but may be combined synergistically with other modalities, such as MAD, weight management, or NMES, to achieve optimal therapeutic control.

New PT technologies continue to advance, including smart bed systems capable of detecting snoring or apnea events and responding automatically by adjusting the bed angle or delivering vibrotactile feedback to prompt postural change [108]. Although still experimental, these innovations reflect the growing emphasis on passive, behavior-modifying treatments that require minimal patient effort once configured. Overall, positional therapy remains a valuable alternative or adjunctive option in POSA, capable of substantially reducing AHI when supine avoidance is maintained. Modern vibratory devices have markedly improved comfort and adherence compared to traditional methods, though randomized controlled trials indicate that some patients may still require supplemental CPAP or other therapies if residual AHI or daytime sleepiness persists above target thresholds.

### 9.3. Other Minimally Invasive Adjuncts

**Figure 10:**
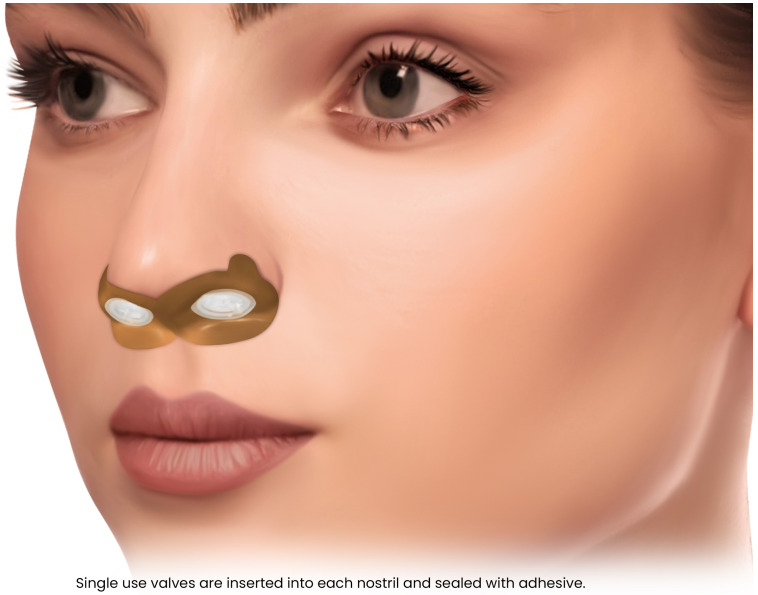
Nasal expiratory positive airway pressure (EPAP) device. Illustration of a single-use nasal EPAP device worn during sleep. Valves inserted into each nostril generate expiratory resistance, creating positive end-expiratory pressure that stabilizes the upper airway and reduces inspiratory collapse in selected patients with obstructive sleep apnea.

A variety of additional minimally invasive therapies can serve as components of a comprehensive management plan for OSA. One such modality is nasal expiratory positive airway pressure (EPAP), which uses small, single-use adhesive valves placed in the nostrils at bedtime (Figure 10). These valves create resistance during exhalation, generating positive end-expiratory pressure that helps maintain upper airway patency during subsequent inspiration. Mechanistically, EPAP functions as a passive analogue of CPAP, providing airway splinting without an external machine or power source. Clinical studies have demonstrated modest but meaningful efficacy, with average reductions in the AHI of approximately 20–30%, particularly among patients with positional or mild-to-moderate OSA [109]. However, response variability is notable, and adherence can be limited by patient discomfort at the adhesive interface or by the sensation of exhaling against resistance. Despite these challenges, EPAP represents an appealing, portable alternative for patients who are CPAP intolerant, especially those with positional or REM predominant OSA. It may also be used as an adjunct to other noninvasive therapies to achieve incremental improvements in AHI. Because EPAP requires no electricity, it is especially convenient for travel or remote environments. The most common side effects are nasal discomfort and congestion, emphasizing the importance of ensuring adequate nasal patency for effective use.

Oropharyngeal myofunctional therapy (MT) involves a series of targeted exercises designed to strengthen and retrain the muscles of the tongue, soft palate, and oropharynx. Typical exercises include tongue-slide and press maneuvers, soft palate blowing, and specific swallowing techniques, often administered under the guidance of speech-language pathologists or physical therapists. Meta-analyses indicate that MT can reduce the AHI by approximately 30% in adults and significantly decrease snoring frequency and intensity [110,111]. The proposed mechanism centers on improved neuromuscular tone and coordination of upper airway dilator muscles, thereby reducing airway collapsibility during sleep. MT is noninvasive, low cost, and essentially risk-free, making it an attractive adjunctive option, especially for patients with mild OSA, primary snoring, or those unable or unwilling to pursue device-based or surgical interventions. The principal limitation is adherence, as meaningful benefit requires consistent daily practice over several months. However, integrating telehealth platforms and mobile applications offers new opportunities to enhance patient engagement and compliance. In contemporary clinical practice, MT is often recommended as a complementary component of multimodal OSA management and may be reasonably encouraged for nearly all motivated patients, given its safety profile and potential functional benefits.

In essence, these minimally invasive adjuncts enable clinicians to develop individualized therapy for patients with OSA. An example of a modern approach might include a patient with moderate POSA who is treated with MAD in combination with a vibratory PT device and performs daily MT. Each component of this regimen targets a distinct physiological contributor to airway collapse, illustrating the potential of precision-based therapy. Future research should systematically evaluate such combination strategies to determine optimal therapeutic pairings, sequencing, and long-term outcomes within the framework of personalized OSA management.

## 10. Pharmacologic Interventions for OSA

Pharmacologic therapy for obstructive sleep apnea (OSA) has historically been limited, reflecting the dominant role of upper airway anatomy in disease pathogenesis. However, contemporary models recognize that OSA severity and persistence are strongly influenced by non-anatomical physiological traits, including obesity-related mechanical load, ventilatory control instability (loop gain), and arousal threshold (Table 13). Advances in understanding these endotypes have enabled the development of targeted pharmacologic strategies that modulate specific contributors to airway collapse. Rather than replacing mechanical therapies, these agents are best conceptualized as disease-modifying or adjunctive interventions within a precision-medicine framework.

### 10.1. Tirzepatide—Weight Loss to Treat OSA

#### 10.1.1. Mechanism of Action

Obesity is one of the most potent and modifiable risk factors for OSA. Excess adiposity increases parapharyngeal and tongue fat volume, reduces lung volumes and caudal traction, and elevates ventilatory demand, collectively increasing upper airway collapsibility. Glucagon-like peptide-1 (GLP-1) receptor agonists and dual GLP-1/glucose-dependent insulinotropic polypeptide (GIP) agonists induce substantial weight loss through appetite suppression, delayed gastric emptying, and metabolic modulation. Reduction in adiposity leads to decreased mechanical loading of the upper airway and improved ventilatory stability during sleep.

#### 10.1.2. Clinical Evidence

Obesity represents one of the most important and modifiable risk factors for OSA. Weight reduction consistently leads to significant improvements in OSA severity and, in some cases, to the cure of this condition. Bariatric surgery is known to markedly reduce AHI, often by >50% and can often resolve OSA after sufficient associated weight loss [112]. Until recently, effective pharmacotherapy for obesity was limited. Tirzepatide, a dual GLP-1 and GIP receptor agonist, is a novel injectable medication for type 2 diabetes and obesity that induces dramatic weight loss, averaging 15–20% of body weight in 72 weeks [113].

Longitudinal observational and randomized interventional evidence consistently demonstrate that changes in body weight are tightly linked to both progression and regression of obstructive sleep apnea, and that the magnitude of weight change determines the degree of disease modification [112].

In the Sleep Heart Health Study, Newman et al. examined the natural history of sleep-disordered breathing over a five-year period in nearly 3000 adults using repeated polysomnography. Weight gain was strongly associated with worsening respiratory disturbance index (RDI), with a clear dose–response relationship. Men who gained more than 10 kg had more than fivefold higher odds of clinically meaningful progression of sleep-disordered breathing compared with those who maintained stable weight (adjusted OR 5.21; 95% CI, 2.35–11.53), while those gaining 5–10 kg also demonstrated substantially increased risk (adjusted OR 2.97; 95% CI, 1.66–5.32). Conversely, substantial weight loss was associated with regression of disease, particularly among men, in whom loss of more than 10 kg was associated with markedly higher odds of improvement (adjusted OR 5.40; 95% CI, 1.69–17.25). Despite these associations, sleep-disordered breathing progressed over time even among participants with stable weight, indicating that weight is a dominant but not exclusive determinant of disease trajectory.

Randomized pharmacologic trials provide interventional confirmation of these observational findings (Table 14). In the SCALE Sleep Apnea trial, Blackman et al. demonstrated that liraglutide 3.0 mg produced a statistically significant reduction in apnea–hypopnea index (AHI) compared with placebo over 32 weeks (estimated treatment difference −6.1 events/h; 95% CI, −11.0 to −1.2), accompanied by moderate weight loss (−5.7%). Post hoc analyses revealed a significant association between the degree of weight loss and improvement in AHI, supporting a causal metabolic contribution to airway obstruction. However, responder-level improvements were modest, and most participants remained within the same OSA severity category at study end.

More recently, two phase-3 randomized controlled trials evaluated tirzepatide in adults with obesity and moderate-to-severe OSA over 52 weeks. Tirzepatide produced large and clinically meaningful reductions in AHI compared with placebo in both participants not receiving positive airway pressure (treatment difference −20.0 events/h; 95% CI, −25.8 to −14.2) and those receiving background PAP therapy (treatment difference −23.8 events/h; 95% CI, −29.6 to −17.9) [22]. These reductions were accompanied by substantial weight loss and significant improvements in hypoxic burden, cardiometabolic markers, and patient-reported sleep outcomes. Across both trials, the magnitude of AHI reduction closely paralleled the degree of weight loss achieved.

Collectively, these data demonstrate a graded continuum of metabolic impact on OSA, ranging from modest disease modification with moderate weight loss to large reductions in disease severity with more potent pharmacologic intervention.

Longitudinal observational data and randomized pharmacologic trials (Table 15) establish adiposity as a causal, modifiable driver of obstructive sleep apnea severity and provide compelling evidence that the magnitude of metabolic intervention determines the extent of clinical benefit. The Sleep Heart Health Study offers foundational natural-history evidence that even modest changes in weight are associated with measurable progression or regression of sleep-disordered breathing, with particularly strong effects observed among men. The finding that OSA frequently progresses despite stable weight underscores the multifactorial nature of disease pathophysiology and explains why weight loss alone rarely normalizes moderate-to-severe OSA.

Pharmacologic trials extend this mechanistic framework into clinical practice. Liraglutide established proof-of-concept that GLP-1 receptor agonism can reduce OSA severity, but the modest magnitude of AHI reduction observed with first-generation agents suggests limited standalone efficacy for many patients with advanced disease. Tirzepatide represents a qualitative advance in metabolic therapy, achieving weight loss of sufficient magnitude to produce AHI reductions approaching those observed with established non-CPAP airway-directed therapies such as hypoglossal nerve stimulation. Importantly, tirzepatide demonstrated benefit both in patients not using PAP therapy and in those receiving background PAP, supporting its role as a complementary disease-modifying intervention rather than a replacement for airway stabilization.

From a precision-medicine perspective, these findings justify the inclusion of a distinct metabolic pathway in OSA treatment algorithms. GLP-1-based therapies are particularly well suited for patients with obesity-predominant OSA, especially those with coexisting cardiometabolic disease, and may be deployed either as primary therapy or as an adjunct to improve eligibility for and outcomes of airway-directed interventions. The sex-specific differences observed in longitudinal data further suggest that biological factors such as fat distribution, airway anatomy, and hormonal milieu may modulate response to metabolic therapy, reinforcing the need for individualized treatment selection.

At the same time, metabolic pharmacotherapy differs fundamentally from definitive anatomic interventions such as maxillomandibular advancement. The durability of OSA improvement with GLP-1-based agents remains dependent on continued therapy and maintenance of weight loss, and discontinuation may be associated with recurrence of disease. These limitations highlight the importance of combination strategies, in which metabolic therapy is integrated with neuromodulatory or structural interventions to address both upstream drivers and downstream airway mechanics.

Overall, the convergence of natural-history and interventional evidence supports a paradigm shift in OSA management: obesity-driven disease should be treated as a modifiable metabolic condition, while recognizing that optimal long-term control in many patients will require a combination of metabolic and airway-directed therapies tailored to underlying pathophysiology.

#### 10.1.3. Clinical Role and Limitations

GLP-1-based metabolic therapies represent the first pharmacologic class to demonstrate consistent, clinically meaningful reductions in OSA severity through disease modification rather than direct airway manipulation. Their primary role is in patients with obesity-predominant OSA, particularly when excess adiposity is a dominant contributor to upper airway collapsibility and when cardiometabolic comorbidity (e.g., type 2 diabetes, hypertension, metabolic syndrome) is present.

These agents may be deployed in several clinically relevant contexts: as primary therapy in patients unwilling or unable to use CPAP; as an adjunctive therapy to reduce residual disease burden in patients treated with CPAP or hypoglossal nerve stimulation; or as a prehabilitation strategy to improve candidacy for airway-directed interventions by lowering body mass index or reducing perioperative risk. The demonstration that tirzepatide reduces AHI substantially even in patients already receiving PAP therapy highlights its potential role in combination treatment paradigms.

However, important limitations must be acknowledged. The durability of OSA improvement is dependent on continued pharmacologic therapy and sustained weight loss, distinguishing metabolic agents from definitive anatomic interventions such as maxillomandibular advancement. Discontinuation is frequently associated with partial or complete weight regain and recurrence of OSA severity. Additionally, while GLP-1-based therapies reduce mechanical load on the airway, they do not directly stabilize pharyngeal collapsibility and are therefore unlikely to normalize OSA in isolation in patients with severe skeletal restriction or neuromuscular collapse. Gastrointestinal adverse effects and cost may further limit long-term adherence in some patients.

### 10.2. Eszopiclone—Increasing Arousal Threshold

#### 10.2.1. Mechanism of Action

A subset of OSA patients exhibits a low respiratory arousal threshold, meaning they awaken in response to relatively minor airway narrowing. While arousal restores airflow, premature awakening prevents sufficient accumulation of respiratory drive to recruit upper airway dilator muscles effectively. This leads to repetitive, short obstructive events and significant sleep fragmentation. Sedative–hypnotic agents such as eszopiclone increase the respiratory arousal threshold by enhancing inhibitory GABAergic signaling in cortical and subcortical arousal pathways. This allows greater negative intrathoracic pressure and hypercapnia to develop before arousal, facilitating: increased activation of the genioglossus and other upper airway dilator muscles, spontaneous airway reopening without cortical arousal, and reduction in event frequency and sleep fragmentation

#### 10.2.2. Clinical Evidence

In a study of OSA patients using eszopiclone 3 mg vs. placebo. Eszopiclone significantly increased the respiratory arousal threshold from −18.0 cm H_2_O in the placebo group to −14.0 cm H_2_O in the Eszopiclone group. This indicates that patients could tolerate greater negative intrathoracic pressure and thus greater respiratory drive before waking [14]. As hypothesized, eszopiclone treatment reduced the AHI compared with placebo, without prolonging apneas or causing oxygen desaturation. The therapeutic effect was most pronounced in the subgroup of patients with a low baseline arousal threshold, in whom AHI decreased from 25 to 14 events per h, resulting in a mean reduction of approximately 40% [14]. Sleep quality and total sleep time also improved significantly in the eszopiclone arm. Importantly, eszopiclone did not exacerbate nocturnal hypoxemia or prolong respiratory events, suggesting that selective elevation of the arousal threshold can stabilize breathing without compromising gas exchange. By preventing premature arousals, eszopiclone allows additional breaths to occur before an apnea-related awakening, thereby improving respiratory stability in patients without severe hypoxemia. While this was a single-night, controlled laboratory study, the findings support the concept that sedative hypnotic therapy may benefit a definable OSA endotype characterized by a low arousal threshold. As a standalone intervention, such treatment may not completely eliminate OSA, but could be further enhanced by combining it with complementary therapies in future studies.

#### 10.2.3. Role and Limitations

Sedative–hypnotic therapy targeting the respiratory arousal threshold occupies a narrow but mechanistically well-defined role in OSA management. These agents are most relevant in patients with low arousal threshold phenotypes, characterized by frequent brief arousals, relatively preserved airway anatomy, and limited ability to recruit upper airway dilator muscles before awakening. In such patients, increasing arousal threshold may permit accumulation of respiratory drive sufficient to reopen the airway without cortical arousal, reducing event frequency and sleep fragmentation. Clinically, arousal-threshold modulation is best considered as an adjunctive therapy rather than monotherapy. For patients with mild to moderate OSA and insomnia or fragmented sleep, a bedtime sedative such as eszopiclone or zolpidem might improve both their insomnia and modestly reduce OSA severity. It may enhance the effectiveness of mechanical treatments such as CPAP, MADs, or positional therapy by improving sleep continuity and reducing arousal-driven instability. In carefully selected patients with mild-to-moderate OSA, sedative therapy may also provide a modest standalone benefit.

A critical pathophysiologic requirement for arousal-threshold modulation is that pharmacologic agents increase the respiratory arousal threshold without reducing upper airway dilator muscle activity. Sedative agents that relax pharyngeal musculature or blunt neuromuscular responsiveness may worsen airway collapsibility, prolong obstructive events, and increase hypoxemia [14,115]. Therefore, this therapeutic strategy is only appropriate when the drug selectively increases cortical arousal threshold while preserving, or at a minimum not suppressing, genioglossus and other upper airway dilator muscle activation. Eszopiclone has been shown in physiological studies to increase the respiratory arousal threshold without significantly impairing upper airway muscle responsiveness or prolonging apneas in carefully selected patients [14]. The absence of upper airway muscle suppression distinguishes it from sedative agents that may exacerbate obstruction. Consequently, arousal-threshold modulation should be restricted to patients with demonstrably low arousal thresholds and adequate neuromuscular compensatory capacity.

Limitations are substantial. Sedative–hypnotics do not address underlying airway obstruction, and indiscriminate use may worsen hypoventilation or hypoxemia in patients with severe anatomic collapse, obesity hypoventilation, or coexisting pulmonary disease. Evidence supporting long-term efficacy remains limited, and interindividual variability in response is high. Accordingly, these agents require careful patient selection, avoidance in high-risk phenotypes, and cautious integration into multimodal treatment plans. Longer-term and larger outcome trials are needed, but early evidence suggests that, if used judiciously, pharmacologically raising the arousal threshold can stabilize breathing and should not be reflexively contraindicated in all OSA patients, as it can be part of an individualized regimen.

### 10.3. Carbonic Anhydrase Inhibitors—Lowering Loop Gain

#### 10.3.1. Mechanism of Action

Ventilatory loop gain reflects the sensitivity of the respiratory control system to perturbations. High loop gain leads to exaggerated ventilatory responses, resulting in oscillations between hyperventilation and apnea. Although classically associated with central sleep apnea, elevated loop gain also contributes to obstructive events by destabilizing breathing and promoting repetitive airway collapse. Carbonic anhydrase inhibitors (CAIs), such as acetazolamide and sulthiame, reduce loop gain through several mechanisms:

By inducing a mild metabolic acidosis via renal bicarbonate loss. The resulting acidemia increases baseline ventilatory drive and typically reduces PaCO_2_, while simultaneously decreasing ventilatory instability by dampening the magnitude of chemoreflex-driven ventilatory responses. By reducing ventilatory overshoot and stabilizing breathing around the apneic threshold, CAIs attenuate cyclical apnea–hyperventilation patterns and can reduce the frequency of obstructive events in patients with ventilatory control instability. As a result of ventilatory stabilization, CAIs lower the propensity for cyclical airway collapse, leading to reductions in apnea frequency and improvements in oxygenation. CAIs do not directly alter airway anatomy but can unmask the effectiveness of existing airway patency by reducing destabilizing ventilatory oscillations. This explains their utility as adjuncts to anatomic or neuromodulatory therapies in patients with persistent respiratory instability. Although CAIs generally lower PaCO_2_ due to increased ventilation, the reduction in loop gain reflects changes in respiratory control stability (response to perturbations) rather than a simple directional change in PaCO_2_.

#### 10.3.2. Clinical Evidence

In a 4-week, double-blind, randomized, placebo-controlled dose-guiding trial of adults with moderate–severe OSA who were intolerant of PAP, Sulthiame produced dose-dependent reductions in OSA severity versus placebo. Nightly 400 mg lowered mean AHI from 55.2 to 33.0 events per h, and 200 mg lowered AHI from 61.1 to 40.6 events per h; placebo changed 53.9 → 50.9 events/h [116]. Responder rates defined as ≥50% AHI reduction were 40% at 400 mg, 25% at 200 mg, and 5% with placebo [116]. Overnight oxygenation improved as well. Patient-reported outcomes, such as sleepiness and sleep quality, did not change significantly over 4 weeks, suggesting that physiologic gains may precede symptomatic benefits or be offset by adverse effects, or that the short trial duration may limit detectable changes. Safety was acceptable overall; paresthesias were common at the 400 mg dose, dyspnea occurred in 18% at that dose, and no serious adverse events were reported. Collectively, these data indicate that STM substantially reduces AHI and modestly improves oxygenation in CPAP-intolerant, moderate-to-severe OSA [116]. The improvements in AHI of about 40% mean reduction at 400 mg are comparable to those from using an oral appliance or moderate weight loss, which is a significant effect size for a drug. It is plausible that, in the future, carbonic anhydrase inhibitors will be prescribed for OSA, particularly for those with ventilatory control issues or exposure to altitude.

Acetazolamide (AZM), a more commonly used CAI, has also shown efficacy in OSA. A meta-analysis comprising 28 studies shows that Acetazolamide significantly reduced AHI by 37.7% (13.8 events per h) and increased oxygen nadir by 4.4%, with similar efficacy across OSA and CSA subtypes [117]. Side effects of AZM include diuresis, fatigue, taste alterations, and kidney stone risk with extended use. Sulthiame’s development was partly to find a CAI with fewer off-target effects for chronic usage in epilepsy, its original indication, and now possibly OSA.

#### 10.3.3. Role and Limitations

Carbonic anhydrase inhibitors (CAIs) provide a direct pharmacologic means of stabilizing ventilatory control by reducing loop gain, making them particularly attractive for patients in whom ventilatory instability is a dominant contributor to OSA. This includes individuals with high chemoresponsiveness, periodic breathing patterns, or mixed obstructive–central features. In such patients, CAIs may substantially reduce apnea burden by dampening oscillations in ventilatory drive. In clinical practice, CAIs are best positioned as adjunctive therapies for patients who remain symptomatic or have residual respiratory instability despite airway-directed interventions. They may also be considered in PAP-intolerant patients with physiologic evidence of high loop gain, where anatomic therapies alone are insufficient. CAIs would likely be used in combination with other therapies. For example, a patient with high loop gain and moderate OSA might not respond well to MAD alone, but adding a CAI could significantly enhance results by preventing hypocapnic central events or overshoot-induced apneas. Importantly, these drugs lower blood pressure slightly and have been used in hypertension, so they might confer cardiovascular benefit as well. The main caution is to ensure tolerability and monitor metabolic effects.

CAIs are limited by dose-dependent adverse effects, including paresthesias, dyspnea, fatigue, and electrolyte disturbances, which can restrict tolerability and long-term use. Their effects on AHI, while meaningful, are generally partial rather than curative, and long-term outcome data—particularly regarding cardiovascular risk reduction—are lacking. As with arousal-threshold modulation, CAIs do not correct structural airway collapse and therefore should be integrated thoughtfully into combination strategies rather than used in isolation for advanced disease.

## 11. Discussion

**Figure 11:**
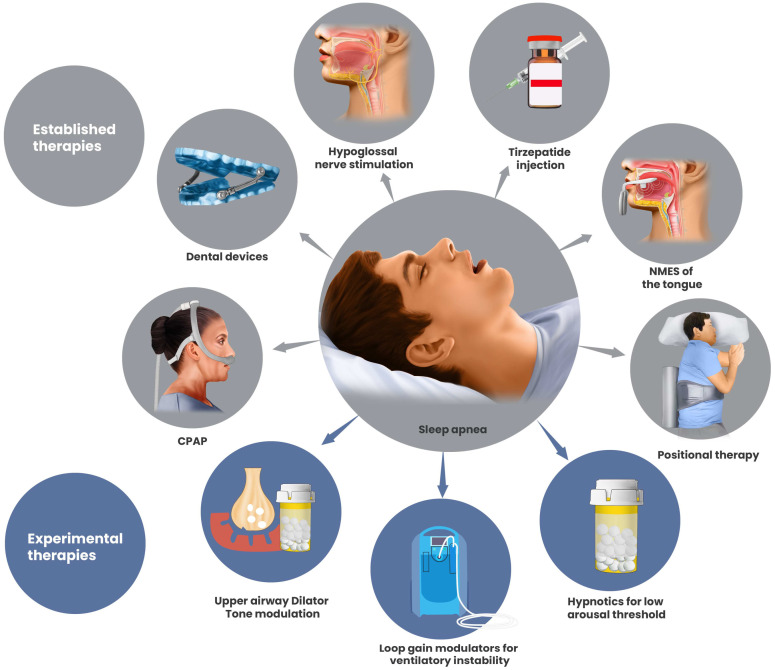
Multimodal therapeutic landscape for obstructive sleep apnea. Schematic illustration depicting established and emerging therapies for obstructive sleep apnea and their complementary roles in disease management. Airway-directed interventions include continuous positive airway pressure (CPAP), mandibular advancement devices, hypoglossal nerve stimulation, neuromuscular electrical stimulation of the tongue, and positional therapy. Pharmacologic and endotype-targeted approaches address non-anatomical contributors to airway collapse, including metabolic modulation with GLP-1-based therapy, ventilatory control instability (loop-gain modulation), and arousal threshold manipulation. Together, these modalities illustrate a precision-guided, multimodal framework for individualized OSA treatment.

The landscape of OSA management is rapidly evolving from a one-size-fits-all model, as in CPAP therapy, to an individualized, multimodal approach. This review highlights a fundamental shift in the management of obstructive sleep apnea (OSA): from a historically CPAP-centric paradigm toward a precision-guided, multimodal framework that aligns treatment with the dominant anatomic and physiological drivers of disease in individual patients. While continuous positive airway pressure remains the most physiologically efficacious therapy when used optimally, real-world adherence limitations necessitate alternative strategies capable of achieving durable disease control across diverse phenotypes (Figure 11). 

Across non-CPAP therapies, our synthesis demonstrates clear heterogeneity in both magnitude and durability of effect. Mandibular advancement devices (MADs) consistently reduce OSA severity relative to placebo and provide symptom relief comparable to CPAP in mild-to-moderate disease, largely due to superior adherence. These findings are concordant with prior systematic reviews and meta-analyses, including Papageorgiou et al., which similarly concluded that MADs represent an effective second-line therapy in appropriately selected patients. However, by incorporating standardized directionality across trials and explicitly distinguishing physiologic efficacy from real-world effectiveness, our review extends prior work by clarifying why MADs often achieve comparable clinical outcomes despite inferior per-night AHI reduction.

Surgical therapies illustrate the importance of matching mechanism to anatomy. Palatal surgery, including UPPP and its variants, provides meaningful short-term improvement in selected patients with retropalatal obstruction, consistent with findings from earlier reviews. However, our synthesis emphasizes a key distinction often under-represented in prior reviews: limited long-term durability. Longitudinal cohort data demonstrate attenuation of benefit over time and, in population-level analyses, inferior survival compared with CPAP. In contrast, maxillomandibular advancement (MMA) stands apart as the most efficacious and durable surgical intervention, achieving reductions in AHI comparable to CPAP and superior outcomes relative to other surgical modalities. While Papageorgiou et al. acknowledged the high efficacy of MMA, our review expands on this by integrating randomized comparisons, real-world effectiveness metrics, and long-term durability data to position MMA as a definitive, adherence-independent therapy for carefully selected patients with skeletal restriction.

Hypoglossal nerve stimulation (HNS) represents a major advance in neuromodulatory therapy for OSA. Consistent with prior systematic reviews, including Papageorgiou et al., we confirm that HNS produces substantial and sustained improvements in AHI, oxygenation, and daytime symptoms with high adherence. Importantly, this review advances the field by integrating randomized responder-based metrics, long-term durability data, and real-world registry outcomes to quantify both magnitude and persistence of benefit. Furthermore, emerging bilateral and proximal-targeted stimulation systems suggest that the therapeutic envelope of HNS may continue to expand, reinforcing its role as a durable alternative for CPAP-intolerant patients with favorable anatomy.

Neuromuscular electrical stimulation (NMES) and positional therapy occupy a distinct niche. Our findings are consistent with prior reviews in demonstrating modest average reductions in respiratory event burden, particularly in mild or positional OSA. However, by separating daytime training-based NMES from nocturnal transcutaneous stimulation and contrasting both with implanted HNS, this review provides greater mechanistic clarity regarding why these therapies should be viewed as adjunctive or early-stage interventions, rather than substitutes for definitive airway stabilization in moderate-to-severe disease.

A major breakthrough highlighted in this review, largely absent from earlier summaries such as Papageorgiou et al., is the emergence of metabolic and endotype-targeted pharmacotherapy as disease-modifying treatment for OSA. Longitudinal observational data establish a dose-dependent relationship between weight change and progression or regression of sleep-disordered breathing, while randomized trials of GLP-1-based therapies demonstrate that pharmacologic weight loss can produce clinically meaningful reductions in OSA severity. The magnitude of AHI reduction observed with dual GLP-1/GIP agonism approaches that of some device-based therapies, marking a qualitative advance beyond earlier weight-loss strategies. Similarly, carbonic anhydrase inhibitors and sedative agents targeting ventilatory loop gain and arousal threshold represent a mechanistically novel class of therapies that directly address non-anatomical contributors to airway instability.

**Figure 12:**
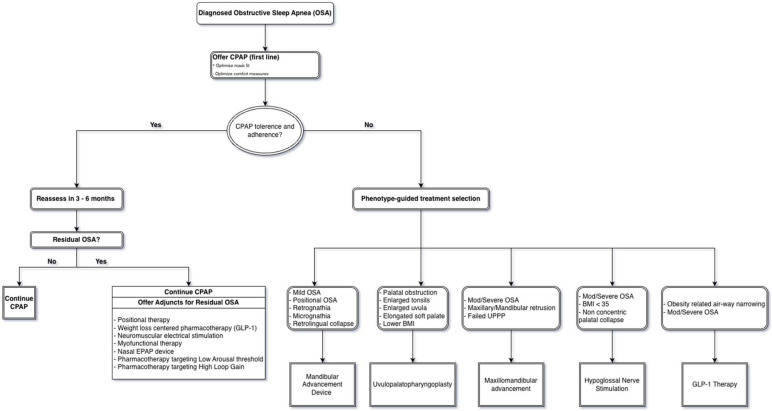
Phenotype-guided selection of therapies for obstructive sleep apnea. Following diagnosis, continuous positive airway pressure (CPAP) is offered as first-line therapy with optimization of mask fit and comfort. Patients are reassessed to evaluate tolerance, adherence, and residual disease. In patients who are intolerant of CPAP or who have residual obstructive sleep apnea, treatment selection is guided by dominant pathophysiologic phenotype, including disease severity, anatomic site of airway collapse, craniofacial structure, body mass index, and prior treatment response. Adjunctive therapies may be combined for residual disease. This framework emphasizes precision-based treatment selection rather than reliance on apnea–hypopnea index severity alone.

Taken together, these data support a model in which no single therapy universally dominates, but rather where optimal management arises from aligning therapy with phenotype—anatomic, neuromuscular, ventilatory control, arousal threshold, and metabolic load (Figure 12). This perspective extends prior reviews by integrating emerging pharmacologic therapies and by emphasizing combination strategies capable of addressing multiple pathophysiologic traits simultaneously (Figure 11).

### 11.1. Economic Considerations and Value-Based Treatment Selection

Economic considerations are increasingly central to treatment selection in obstructive sleep apnea, particularly as therapeutic options expand beyond continuous positive airway pressure. While cost is often framed in terms of upfront expense, the true economic impact of OSA therapies is determined by a combination of initial cost, durability of benefit, adherence, and downstream health outcomes. As such, cost-effectiveness in OSA cannot be evaluated in isolation from clinical effectiveness and long-term treatment persistence.

Continuous positive airway pressure therapy has the lowest upfront cost and is widely covered by insurance, making it the most accessible treatment option. When adherence is sustained, CPAP offers exceptional physiologic efficacy and favorable cost-effectiveness by reducing cardiovascular risk and healthcare utilization. However, real-world adherence is frequently suboptimal, and discontinuation or inconsistent use substantially diminishes both clinical benefit and economic value. In this context, low acquisition cost does not necessarily translate into optimal value for all patients.

Mandibular advancement devices occupy an intermediate economic position. Although their upfront cost exceeds that of CPAP, MADs demonstrate high long-term adherence, particularly in patients with mild-to-moderate disease. Multiple cost-effectiveness analyses suggest that, in appropriately selected patients, MADs may provide comparable value to CPAP by achieving sustained symptom relief and quality-of-life improvement despite higher residual AHI. This highlights the importance of incorporating adherence into economic evaluations rather than relying solely on physiologic efficacy.

Surgical and implantable therapies present the most complex economic trade-offs. Maxillomandibular advancement and hypoglossal nerve stimulation are associated with high upfront costs, often requiring extensive preauthorization and patient selection. However, both therapies provide durable, adherence-independent benefits. For MMA, long-term efficacy and absence of ongoing treatment costs may offset initial expense over time, particularly in patients with severe disease who would otherwise require lifelong PAP therapy. Similarly, although HNS carries one of the highest initial costs among OSA therapies, economic modeling suggests that its value improves substantially over a 5–10 year horizon due to high adherence, durable efficacy, and reductions in OSA-related morbidity. These considerations are particularly relevant for younger patients with long-anticipated treatment durations.

Emerging pharmacologic therapies introduce a distinct economic profile. GLP-1-based agents incur high ongoing costs, and insurance coverage remains variable, particularly when prescribed for OSA rather than obesity or diabetes. However, these therapies may deliver value beyond OSA-specific outcomes by simultaneously reducing cardiometabolic risk, healthcare utilization, and long-term complications of obesity. When such broader benefits are considered, the cost–benefit balance may be more favorable than suggested by OSA outcomes alone. Nevertheless, the need for continuous therapy and the risk of disease recurrence with discontinuation remain important economic limitations.

Lower-cost adjunctive therapies, including positional devices, nasal EPAP, neuromuscular electrical stimulation, and pharmacologic modulation of arousal threshold or ventilatory control, generally provide modest clinical benefit at relatively low cost. Their economic value is greatest when used in carefully selected patients or as part of combination strategies that enhance the effectiveness of primary therapies rather than as standalone treatments.

Table 16 illustrates a value-based, precision-guided overview of OSA management in which economic considerations are integrated with disease severity, pathophysiology, and patient preference. Rather than favoring the least expensive therapy or the most efficacious intervention in isolation, optimal treatment selection balances cost, durability, adherence, and long-term health impact. As the therapeutic landscape continues to evolve, future cost-effectiveness analyses incorporating real-world adherence and long-term outcomes will be essential to guide sustainable, patient-centered care.

### 11.2. Limitations and the Need for Long-Term Safety and Durability Data

Despite substantial advances in the treatment of obstructive sleep apnea (OSA), a major limitation across many emerging and alternative therapies is the lack of robust long-term safety and durability data extending beyond five years. While short- and intermediate-term studies demonstrate meaningful improvements in respiratory indices, symptoms, and quality of life, the long-term clinical implications of chronic therapy exposure, device implantation, or sustained pharmacologic modulation remain incompletely defined.

#### 11.2.1. Device-Based and Surgical Therapies

For hypoglossal nerve stimulation (HNS), prospective trials and large registries provide reassuring efficacy and safety data through five years, including stable reductions in apnea–hypopnea index (AHI), sustained improvements in patient-reported outcomes, and acceptable device-related adverse event rates [19,89]. However, experience beyond this time horizon is limited. Important unanswered questions remain regarding long-term lead integrity, battery longevity, revision rates, and the cumulative effects of chronic neuromodulation on nerve and muscle function, particularly as candidacy broadens and newer stimulation paradigms are introduced.

Similarly, neuromuscular electrical stimulation (NMES) devices have demonstrated favorable short-term tolerability and modest efficacy in selected patients, but available studies are limited to weeks or months of follow-up [97]. Data on long-term adherence, sustained neuromuscular adaptation, potential desensitization, and downstream clinical outcomes are lacking, and no studies have evaluated outcomes beyond one year. Consequently, NMES should currently be regarded as an adjunctive or early-stage intervention rather than a definitive long-term therapy.

For surgical interventions, durability varies substantially by procedure. Maxillomandibular advancement (MMA) benefits from decades of follow-up data demonstrating durable reductions in AHI and sustained symptom improvement [23]. In contrast, isolated palatal surgeries such as uvulopalatopharyngoplasty show attenuation of benefit over time and less consistent long-term outcomes, findings that have been consistently reported in systematic reviews [10]. This heterogeneity in durability highlights the importance of procedure selection and patient counseling.

#### 11.2.2. Pharmacologic and Endotype-Targeted Therapies

Emerging pharmacologic therapies introduce additional long-term uncertainties. GLP-1-based metabolic agents have extensive safety data in populations with obesity and type 2 diabetes; however, their application specifically to OSA raises distinct questions regarding the durability of OSA improvement, the consequences of long-term therapy continuation, and the effects of treatment discontinuation. Available evidence indicates that reductions in OSA severity are closely tied to sustained weight loss, suggesting a risk of disease recurrence with weight regain. Moreover, long-term effects on cardiovascular and neurocognitive outcomes in OSA populations have not yet been fully characterized. Pharmacologic modulation of arousal threshold and ventilatory loop gain is supported primarily by short-term physiological and proof-of-concept studies [14]. While these agents demonstrate mechanistic plausibility and measurable reductions in apnea burden in selected endotypes, data on long-term safety, tolerance, and effectiveness remain sparse. Potential risks include unintended effects on hypoventilation, hypoxemia, and sleep architecture, particularly in patients with severe OSA or comorbid pulmonary disease.

#### 11.2.3. Implications for Clinical Practice and Research

These limitations underscore the importance of cautious integration of emerging therapies into clinical practice. While innovation has expanded the therapeutic armamentarium for OSA, established treatments such as CPAP and MMA continue to benefit from the most extensive long-term evidence. Emerging therapies should therefore be incorporated within a precision-guided, multimodal framework, often as adjuncts or alternatives in carefully selected patients, until longer-term outcomes are better defined.

Future research priorities include extended post-marketing surveillance, long-term registries, pragmatic trials with broader inclusion criteria, and studies evaluating cardiovascular and neurocognitive outcomes. Addressing these gaps will be essential to determining whether short-term improvements translate into durable, clinically meaningful benefits over the lifespan of patients with obstructive sleep apnea.

#### 11.2.4. Limitations of This Review

This review has several important limitations that should be acknowledged. First, the present work is a narrative, structured synthesis rather than a formal systematic review or meta-analysis. Although we employed predefined inclusion criteria and prioritized randomized controlled trials and high-quality observational studies, literature selection was not conducted using a fully PRISMA-compliant methodology. As such, the review may be subject to selection bias, and effect magnitudes reported across modalities reflect the synthesis of representative trials rather than pooled quantitative estimates.

Second, substantial heterogeneity exists across the included studies. Variations in study design, patient selection, OSA severity, outcome definitions, follow-up duration, and reporting standards limit direct comparability between therapies. For example, apnea–hypopnea index reductions are reported using differing baseline severities and response thresholds, while patient-reported outcomes such as the Epworth Sleepiness Scale and quality-of-life measures are inconsistently measured across trials. This heterogeneity constrains the ability to draw definitive head-to-head conclusions and underscores the need for standardized comparative trials.

Third, the absence of formal meta-analytic pooling precludes the generation of summary effect sizes with weighted precision across all therapies. While we provide representative effect estimates from key randomized trials, these values should be interpreted as illustrative rather than definitive comparative metrics.

Fourth, emerging therapies, including hypoglossal nerve stimulation, neuromuscular electrical stimulation, and novel pharmacologic intervention, are supported primarily by short- to intermediate-term data. Long-term durability, safety, and cardiovascular outcome effects remain incompletely characterized, which may influence future comparative assessments.

Finally, economic comparisons presented in this review are derived from published estimates and cost-effectiveness analyses but do not represent formal economic modeling performed within this study. Costs may vary substantially by healthcare system, payer structure, and geographic region.

Despite these limitations, this review integrates contemporary evidence across established and emerging therapies within a mechanistically informed framework. By organizing treatment modalities according to pathophysiologic targets and clinical phenotypes, we aim to provide a structured and clinically relevant synthesis that supports precision-guided decision-making in adult obstructive sleep apnea.

### 11.3. Future Studies and Recommendations

Combination Therapy Trials: Conduct RCTs comparing combined treatments to single modality treatments. For example, compare CPAP alone vs. CPAP plus sedative in low-arousal-threshold patients for improvements in adherence and outcomes, or MAD vs. MAD plus weight loss drug in obese OSA for incremental benefit. The goal is to see if multi-target therapy yields additive or synergistic improvements.

Cost-Effectiveness Analyses: As healthcare systems consider covering expensive interventions such as HNS surgery or lifelong drug therapy, robust cost–benefit analyses are needed. These should incorporate not only OSA severity reduction but also quality-of-life gains and potential savings from avoided comorbid events, such as fewer hypertension medications or fewer diabetes complications if weight loss is achieved. Early modeling suggests that HNS is cost-effective in patients with CPAP failures and moderate-to-severe OSA. Similar analyses will be required for pharmacotherapies, which may entail ongoing costs.

Refining Patient Selection Tools: Development of easily accessible tests, perhaps via short questionnaires or simplified home measurements to estimate a patient’s OSA endotypic traits, would greatly aid clinicians. Work is ongoing to use airflow patterns from diagnostic sleep studies to infer loop gain or an arousal threshold. If validated, such tools can become part of a routine sleep report. Such data could be useful to physicians, as they can identify patients likely to have high loop gain and be good candidates for CAI therapy. This will require training sleep specialists to be comfortable prescribing and monitoring these medications, shifting the focus away from purely device-centric therapy.

Continued Emphasis on CPAP Improvement: Even as alternatives flourish, CPAP will remain a mainstay, especially for severe OSA or for those who prefer it and do well with it. Research shouldn’t abandon CPAP innovation. Future development should continue to advance new interface designs, improved humidification, more advanced algorithms to anticipate patient breathing, and integration with other technologies, which could further mitigate CPAP’s downsides. Moreover, educational and behavioral support via telehealth monitoring could improve CPAP adherence rates closer to the ideal. The competition from other therapies will hopefully spur CPAP manufacturers to enhance user-friendliness.

## 12. Conclusions

The management of OSA continues to evolve with ever-increasing therapeutic possibilities. No longer is CPAP the only answer; instead, we now have a spectrum of effective treatments, from oral appliances and upper-airway surgeries to neurostimulation devices, muscle training tools, and pharmacologic agents, each with unique benefits that can be matched to patient needs. The onus is on clinicians to stay informed of these advances and practice multidisciplinary, personalized care. For patients, this expansion means more hope for a therapy they can live with, ultimately leading to better long-term adherence and better health outcomes. As the field progresses, the OSA patient of the future will likely undergo a comprehensive evaluation and receive a tailored combination of interventions, perhaps including a weight-loss medication, a custom oral appliance, a nighttime positional buzzer, and some throat exercises for good measure. CPAP, of course, remains an available option for those poor responders to less invasive interventions.

Realizing this vision will require continued research, patient education, and, often, a combination strategy addressing both the cause and the consequence. The advances in treatment, from PAP to pills, are closing the gap toward making OSA a controllable and, in some cases, a reversible condition for most patients. The next decade will likely bring even more integration of these therapies, guided by data on what improves not just the AHI but the patient’s life and long-term health. By embracing a multimodal approach now and refining it with future evidence, clinicians can ensure that the right treatment, or mix of treatments, is delivered to the right patient at the right time, thereby fulfilling the ultimate goal of OSA management: effective therapy that patients will actually use.

## Figures and Tables

**Table 5:** Randomized and comparative studies evaluating UPPP against non-surgical management and CPAP, reporting objective respiratory indices, symptom outcomes, and survival where available [58,64].

Study	Design	N (UPPP/Comparator)	Comparator	Outcome	Time Point	Effect Estimate	Interpretation
MacKay et al., 2020 (SAMS) [64]	Multicenter parallel-group RCT	51	Medical management	AHI (events/h)	6 months	MD −17.6 (95% CI −26.8 to −8.4)	UPPP superior
	Multicenter parallel-group RCT	51	Medical management	ESS (points)	6 months	MD −6.7 (95% CI −8.2 to −5.2)	UPPP superior
Sommer et al., 2016 [58]	Randomized controlled trial	18	Baseline	AHI (events/h)	3 months	MD −11.2 (*p* = 0.036)	UPPP superior

Footnotes: Sommer et al., 2016 [58]: Effect estimate represents the between-group difference in mean change from baseline (difference-in-differences). The study reported within-group confidence intervals but did not provide a 95% confidence interval for the between-group contrast; therefore, only the point estimate and *p*-value are shown. Abbreviations: AHI, apnea–hypopnea index; ESS, Epworth Sleepiness Scale; UPPP, uvulopalatopharyngoplasty.

**Table 6:** Uncontrolled and long-term durability studies highlighting short-term efficacy and long-term relapse patterns [65,66].

Study	Design	N	Comparator	Outcome	Time Point	Effect Estimate	Interpretation
Lundkvist et al. [66]	Prospective cohort	158	Baseline	ODI (events/h)	1 year	Median −15 (23 → 8), *p* < 0.001	Significant improvement
Sundman et al., 2021 [65]	Prospective intervention	65	Baseline	AHI (events/h)	2 years	Mean −26.1 (41.6 → 15.5)	Large short-term benefit
				AHI (events/h)	8 years	Mean +1.6 (41.6 → 43.2), *p* = 0.80	No sustained benefit
				AHI (events/h)	2 → 8 years	Mean +27.7, *p* < 0.001	Significant relapse

Footnotes: Uncontrolled studies: Effect estimates reflect within-group pre–post changes. No concurrent comparators were available; therefore, between-treatment comparisons and confidence intervals versus CPAP or other modalities could not be calculated. Abbreviations: AHI, apnea–hypopnea index; ODI, oxygen desaturation index.

**Table 7:** Controlled and comparative studies evaluating MMA against CPAP/APAP and other surgical modalities, emphasizing physiologic efficacy and relative effectiveness [17,79,80].

Study	Design	N (MMA/Comparator)	Comparator	Outcome	Time Point	Effect Estimate	Interpretation
Riley et al., 1990 [80]	Prospective within-patient comparison	30	CPAP	RDI (events/h)	Post-treatment	MMA − CPAP = +0.2	Comparable physiologic efficacy
Vicini et al., 2010 [79]	Randomized controlled trial	25/25	APAP	AHI improvement	Post-treatment	No significant difference (*p* = 0.21)	Comparable efficacy
				ESS improvement	Post-treatment	No significant difference (*p* = 0.20)	Comparable symptom improvement
Boyd et al., 2013 [17]	Retrospective comparative cohort	37/34	UPPP	AHI (events/h)	Post-surgery	MD −21.1 (baseline-adjusted; *p* < 0.0001)	MMA superior
		37/35	UPPP + MMA	AHI	Post-surgery	No difference vs. MMA (*p* = 0.684)	Palatal add-on not beneficial

Footnotes: Riley et al., 1990 [80]: Within-patient physiologic comparison; confidence intervals for MMA–CPAP differences were not reported. Vicini et al., 2010 [79]: Between-group comparisons of improvement in AHI and ESS were formally tested and found not to be statistically different (*p* = 0.21 and *p* = 0.20, respectively); confidence intervals were not reported. Boyd et al., 2013 [17]: Effect estimates represent baseline-adjusted differences in mean change; confidence intervals for between-group contrasts were not reported. Abbreviations: AHI, apnea–hypopnea index; RDI, respiratory disturbance index; ESS, Epworth Sleepiness Scale; MMA, maxillomandibular advancement; UPPP, uvulopalatopharyngoplasty.

**Table 8:** Prospective, real-world, and mechanistic studies highlighting magnitude of effect and cure rates [81,82].

Study	Design	N	Comparator	Outcome	Time Point	Effect Estimate	Interpretation
Rubio-Bueno et al., 2017 [82]	Prospective cohort	34	Baseline	AHI (events/h)	Post-op	Mean −31.8 (38.3 → 6.5), *p* < 0.001	Large reduction
				ESS (points)	Post-op	Mean −16.6 (17.4 → 0.8), *p* < 0.001	Marked symptom improvement
				Cure rate (AHI < 5)	Post-op	52.90%	High cure proportion
Diecidue et al., 2024 [81]	Retrospective cohort	119	CPAP, MAD, UAS	Mean Disease Alleviation (%)	Post-op	MMA 39.4% (*p* < 0.001)	Highest real-world effectiveness
				Remaining AHI	Post-op	10.5 ± 11.8	Low residual disease

Footnotes: Rubio-Bueno et al., 2017 [82]: Effect estimates reflect within-group pre–post changes; no concurrent comparator was included. Diecidue et al., 2024 [81]: Mean Disease Alleviation integrates treatment efficacy and adherence and reflects real-world effectiveness rather than physiologic efficacy alone. Abbreviations: AHI, apnea–hypopnea index; ESS, Epworth Sleepiness Scale; MMA, maxillomandibular advancement; UAS, upper airway stimulation.

**Table 10:** Randomized treatment–control effect estimates for hypoglossal nerve stimulation from the THN3 trial. Outcomes are reported using binary responder-based comparative metrics, including relative risk (RR), odds ratio (OR), absolute risk difference (ARD), and number needed to treat (NNT), with corresponding 95% confidence intervals.

NNT	95% CI (NNT)	Endpoint	Treatment Responders	Control Responders	Risk Difference (ARD)	95% CI (ARD)	Relative Risk (RR)	95% CI (RR)	Odds Ratio (OR)	95% CI (OR)
3.1	2.3–4.6	AHI responder (≥50% reduction and AHI ≤ 20)	72/138 (52.3%)	27/138 (19.6%)	0.326	0.220–0.433	2.67	1.83–3.88	4.48	2.62–7.67

Footnotes: Responder definitions were prespecified: AHI response defined as ≥50% reduction from baseline and AHI ≤ 20 events/h; ODI response defined as ≥25% reduction from baseline. Effect estimates reflect randomized treatment–control comparisons at Month 4. NNT values are reported only where the ARD confidence interval does not cross zero. Longer-term responder proportions (12–15 months) reflect pooled active-therapy performance and are not direct randomized comparisons.

**Table 11:** Randomized evidence evaluating neuromuscular electrical stimulation in obstructive sleep apnea, including short-term changes in respiratory event burden, symptoms, adherence, and safety. NMES effects are modest in magnitude and position-dependent, consistent with a neuromuscular conditioning mechanism [97].

Study	Design	N (Active/Control)	Comparator	Outcome	Time Point	Effect Estimate	Interpretation
Abreu et al., 2023 [97]	Randomized, double-masked, sham-controlled trial	21/19	Sham NMES	REI	6 weeks	−3.3 ± 0.9 (−32.7%; 95% CI 15.5–49.9%)	Significant reduction vs. sham
				Supine REI	6 weeks	Greater reduction than non-supine	Position-dependent effect
				ESS	6 weeks	Improved with active, not sham	Symptom benefit
				Adherence	6 weeks	>90%	Excellent tolerability
				Serious adverse events	6 weeks	None	Favorable safety

Footnotes: Population limitation: NMES was studied in mild OSA; findings are not generalizable to moderate–severe disease. Outcome definition: REI was used instead of AHI; values are not directly comparable with PAP, HNS, or MMA outcomes. Comparability: NMES differs fundamentally from hypoglossal nerve stimulation (daytime neuromuscular conditioning vs. nocturnal real-time airway stabilization) and should be interpreted as an adjunctive therapy. Duration: Outcomes reflect short-term (6-week) follow-up only.

**Table 12:** Neuromuscular electrical stimulation strategies for adult obstructive sleep apnea. This table summarizes noninvasive and implantable neuromuscular electrical stimulation (NMES) approaches for adult obstructive sleep apnea, including stimulation targets, mechanism of action, clinical evidence, typical patient selection, and key limitations. Strategies are categorized by delivery method (daytime transoral NMES, nocturnal transcutaneous stimulation, and implantable hypoglossal nerve stimulation) to distinguish differences in mechanism, durability, and magnitude of apnea–hypopnea index reduction. The table reflects adult populations and is intended to contextualize NMES within a broader multimodal, phenotype-guided treatment framework [19,94,95,97,100,101,102].

Approach	Timing of Stimulation	Primary Target and Mechanism	Typical Candidate Population	Key Limitations and Considerations
Intraoral Neuromuscular Electrical Stimulation (NMES)	Daytime training during wakefulness	Peripheral stimulation of tongue musculature to enhance baseline muscle tone, endurance, and responsiveness, reducing nocturnal airway collapsibility	Patients with primary snoring or mild obstructive sleep apnea; individuals seeking a noninvasive, daytime therapy	Modest effect on AHI; requires consistent daily use over several weeks; long-term durability and adherence outside trial settings remain uncertain
Transcutaneous Neuromuscular Electrical Stimulation (NMES)	Continuous or cyclic stimulation during sleep	Surface electrode stimulation of submental and suprahyoid muscles to dynamically augment airway patency during sleep	Selected patients with mild-to-moderate OSA who are CPAP intolerant	Variable efficacy; limited ability to precisely target deep musculature; potential discomfort or skin irritation; evidence base remains evolving
Implanted Hypoglossal Nerve Stimulation (HNS)	Inspiratory-synchronized stimulation during sleep	Direct activation of hypoglossal nerve branches innervating tongue protrusor muscles, stabilizing the retrolingual airway	Carefully selected CPAP-intolerant patients with moderate-to-severe OSA, favorable anatomy, and absence of complete concentric palatal collapse	Requires surgical implantation; high upfront cost; eligibility restricted by BMI and airway collapse pattern; long-term cardiovascular outcome data still limited

**Table 13:** Emerging pharmacologic therapies targeting physiological endotypes in adult obstructive sleep apnea. This table summarizes emerging pharmacologic strategies for adult obstructive sleep apnea, organized according to their primary physiological target, including metabolic load (GLP-1/GLP-1–GIP receptor agonists), ventilatory control instability (carbonic anhydrase inhibitors), and arousal threshold modulation (non-benzodiazepine hypnotics). For each therapy, the table outlines the mechanism of action, expected effect on apnea–hypopnea index (AHI), typical candidate phenotype, and key safety considerations. Therapies are presented as adjunctive or phenotype-specific interventions rather than universal alternatives to airway-directed treatment. The table reflects evidence derived primarily from randomized controlled trials and mechanistic physiological studies in adult populations.

Agent	Primary Physiologic Target	Mechanism of Action	Observed Effects on OSA Severity	Key Limitations and Considerations
Tirzepatide (GLP-1/GIP receptor agonist)	Obesity-related anatomical collapsibility and ventilatory load	Induces substantial weight loss through appetite suppression and metabolic modulation, reducing parapharyngeal and tongue fat, upper-airway collapsibility, and ventilatory demand	Large, consistent reductions in AHI (≈20–30 events·h^−1^) and hypoxic burden in adults with moderate–severe OSA and obesity; improvements occur both with and without background PAP therapy	Gastrointestinal adverse effects common; long-term durability dependent on continued therapy; efficacy primarily in obesity-driven OSA phenotypes
Eszopiclone (non-benzodiazepine hypnotic)	Low respiratory arousal threshold	Enhances GABAergic inhibition, increasing arousal threshold and allowing greater ventilatory drive and airway muscle activation before awakening	Modest to moderate AHI reduction (≈30–40%) in selected patients with low baseline arousal threshold; improves sleep continuity without worsening hypoxemia in short-term studies	Benefit limited to specific endotypes; requires careful patient selection; long-term safety and efficacy data in OSA are limited
Sulthiame (carbonic anhydrase inhibitor)	High ventilatory loop gain	Induces mild metabolic acidosis, increasing baseline ventilation and reduction in arterial CO_2_, thereby stabilizing ventilatory control and reducing apnea–hyperventilation cycles	Dose-dependent reductions in AHI (≈30–40%) and modest improvements in oxygenation in moderate–severe OSA; physiologic effects may precede symptomatic benefit	Paresthesias and dyspnea at higher doses; symptom improvement inconsistent; long-term tolerability and cardiovascular outcome data needed

**Table 14:** Randomized controlled trial evidence for GLP-1-based pharmacologic therapy in obstructive sleep apnea. This table summarizes randomized, placebo-controlled trials evaluating GLP-1-based pharmacologic weight-loss therapy in adults with obesity and moderate-to-severe obstructive sleep apnea. Reported outcomes include between-group differences in apnea–hypopnea index (AHI) change and weight loss at prespecified follow-up intervals. Trials differ in duration, background therapy, and baseline positive airway pressure use. Effect estimates are presented as treatment–control differences with 95% confidence intervals where available and reflect metabolic modification of OSA severity rather than direct airway stabilization [22,114].

Study	Design	Population	Time Point	Primary Endpoint	Active Therapy	Placebo	Treatment Effect (95% CI)	Interpretation
Blackman et al., 2016 (SCALE Sleep Apnea) [114]	RCT, double-blind	Obesity + mod–sev OSA; unable/unwilling CPAP	32 weeks	AHI change (events/h)	Liraglutide 3.0 mg: −12.2 ± 1.8	−6.1 ± 2.0	−6.1 (−11.0 to −1.2); *p* = 0.015	Modest reduction
			32 weeks	Weight change (%)	−5.7%	−1.6%	−4.2% (−5.2 to −3.1)	Metabolic effect
Malhotra et al., 2024 (SURMOUNT-OSA 1) [22]	Phase 3 RCT	Obesity + mod–sev OSA; not on PAP	52 weeks	AHI change (events/h)	Tirzepatide: −25.3 (−29.3 to −21.2)	−5.3 (−9.4 to −1.1)	−20.0 (−25.8 to −14.2); *p* < 0.001	Large reduction
Malhotra et al., 2024 (SURMOUNT-OSA 2) [22]	Phase 3 RCT	Obesity + mod–sev OSA; on PAP	52 weeks	AHI change (events/h)	Tirzepatide: −29.3 (−33.2 to −25.4)	−5.5 (−9.9 to −1.2)	−23.8 (−29.6 to −17.9); *p* < 0.001	Large additive effect

Footnotes: Effect estimates represent between-group differences (active therapy minus placebo) derived from randomized, double-blind controlled trials. The SCALE Sleep Apnea trial evaluated liraglutide 3.0 mg as an adjunct to diet and exercise over 32 weeks in participants unwilling or unable to use continuous positive airway pressure therapy. The SURMOUNT-OSA trials evaluated tirzepatide over 52 weeks in participants with obesity and moderate-to-severe obstructive sleep apnea, stratified by baseline use of positive airway pressure therapy. Confidence intervals and *p*-values are as reported in the original publications; no pooled or post hoc statistical analyses were performed. Responder analyses in the liraglutide trial were exploratory and not powered as primary endpoints. Abbreviations: AHI, apnea–hypopnea index; CI, confidence interval; PAP, positive airway pressure; RCT, randomized controlled trial.

**Table 15:** Longitudinal association between weight change and progression or regression of sleep-disordered breathing. This table summarizes sex-stratified, multivariable-adjusted odds ratios for progression and regression of sleep-disordered breathing, measured by changes in respiratory disturbance index (RDI) over a 5-year interval in the Sleep Heart Health Study. Weight change is categorized relative to stable weight and examined across multiple thresholds of RDI worsening and improvement. Odds ratios are adjusted for age, race, baseline weight, and average RDI. Results reflect observational associations and characterize the natural history of disease progression and regression in relation to weight change [112].

Weight Change	Men OR (95% CI)	Women OR (95% CI)
Gain 5–10 kg	2.97 (1.66–5.32)	1.11 (0.50–2.46)
Gain > 10 kg	5.21 (2.35–11.53)	2.55 (0.99–6.56)
Loss > 10 kg	5.40 (1.69–17.25)	1.86 (0.48–7.15)

Footnotes: Respiratory disturbance index (RDI) was measured by polysomnography at baseline and at 5-year follow-up. Odds ratios (ORs) represent the likelihood of progression or regression of sleep-disordered breathing relative to stable weight and are adjusted for age, race, baseline weight, and average RDI, as reported by the original investigators. Stable weight serves as the reference category (OR = 1.00). Weight change categories reflect absolute change in body weight over the follow-up interval. Results represent observational associations and do not imply causation. Abbreviations: OR, odds ratio; CI, confidence interval; RDI, respiratory disturbance index.

**Table 16:** Economic considerations and value-based comparison of obstructive sleep apnea therapies. This table summarizes approximate upfront and ongoing costs, typical insurance coverage patterns, and qualitative cost–benefit considerations for major therapies used in the management of obstructive sleep apnea. Cost estimates reflect commonly reported U.S. ranges derived from published economic analyses, payer coverage policies, and guideline-based sources. Cost–benefit considerations incorporate treatment durability, adherence, need for ongoing therapy, and potential downstream health benefits rather than formal cost-effectiveness modeling. Values are intended for comparative context and may vary by healthcare system, payer, and patient characteristics.

Therapy	Approximate Upfront Cost (USD)	Ongoing Cost	Typical Insurance Coverage	Cost–Benefit Considerations
CPAP/APAP	$500–$2000	$300–$800/year (supplies)	Widely covered (Medicare & commercial); adherence requirements common	Lowest upfront cost; high physiologic efficacy, but cost-effectiveness reduced by poor long-term adherence in some patients
Mandibular advancement device (MAD)	$1800–$3500	$200–$400/year (adjustments/replacement)	Often covered with documentation of CPAP intolerance	Moderate cost with high adherence; favorable cost–benefit in mild–moderate OSA despite residual AHI
Uvulopalatopharyngoplasty (UPPP)	$6000–$10,000	Minimal	Generally covered if criteria met	Lower upfront cost than advanced surgery but limited durability reduces long-term value
Maxillomandibular advancement (MMA)	$20,000–$40,000	Minimal	Covered in selected cases; prior authorization common	High upfront cost but durable, CPAP-level efficacy; favorable long-term cost–benefit in severe OSA
Hypoglossal nerve stimulation (HNS)	$30,000–$45,000	Battery replacement at ~10–12 yrs	Increasingly covered; strict eligibility criteria	Very high initial cost offset by durable efficacy and high adherence; long-term value improves beyond 5–10 years
Neuromuscular electrical stimulation (NMES)	$1000–$2500	Minimal	Often not covered; out-of-pocket common	Low cost but modest efficacy; cost-effective mainly as adjunct or in mild disease
Positional therapy devices	$200–$1000	Minimal	Variable; often limited coverage	Low cost; cost-effective in positional OSA but limited in moderate–severe disease
Nasal EPAP devices	$50–$100/month	Continuous	Variable; often patient-paid	Moderate cumulative cost; useful as backup/travel option rather than primary therapy
GLP-1/GLP-1–GIP therapy	$900–$1300/month	Continuous	Variable; improving for obesity/diabetes	High ongoing cost; cost–benefit improves with cardiometabolic risk reduction and durable weight loss
Arousal-threshold modulators	$10–$50/month	Continuous	Typically covered	Low cost but limited standalone efficacy; adjunctive role
Carbonic anhydrase inhibitors	$10–$40/month	Continuous	Covered	Low cost; moderate benefit in selected high loop-gain phenotypes

Footnotes: Cost estimates were derived from published economic analyses, clinical guidelines, payer coverage determinations, and large registry studies: Refs. [9,118,119,120,121,122,123] Values represent approximate U.S. ranges and are intended for comparative context rather than precise cost accounting”. Insurance coverage assumes documentation of medical necessity and may require prior authorization or demonstration of CPAP intolerance. Cost estimates represent approximate U.S. ranges synthesized from published cost-effectiveness analyses, clinical guidelines, and payer coverage determinations. Values are provided for comparative context rather than precise pricing and may vary substantially by region, payer, device manufacturer, and healthcare system.

## Data Availability

This manuscript is a narrative review based on previously published literature. No new datasets were generated or analyzed during the current study.
